# Phytochemistry and Pharmacological Activities of the Diterpenoids from the Genus *Daphne*

**DOI:** 10.3390/molecules26216598

**Published:** 2021-10-31

**Authors:** Yi-Wen Nie, Yuan Li, Lan Luo, Chun-Yan Zhang, Wei Fan, Wei-Ying Gu, Kou-Rong Shi, Xiao-Xiang Zhai, Jian-Yong Zhu

**Affiliations:** 1 Department of Pharmacy, Seventh People’s Hospital Affiliated to Shanghai University of Traditional Chinese Medicine, Shanghai 200137, China; shan121488@hotmail.com (Y.-W.N.); yuan_liyy@126.com (Y.L.); fanwei8106@163.com (W.F.); pdguweiying@163.com (W.-Y.G.); shikourong@163.com (K.-R.S.); 2 Central Laboratory, Seventh People’s Hospital Affiliated to Shanghai University of Traditional Chinese Medicine, Shanghai 200137, China; zhcy205@163.com; 3 Department of Dermatology, Seventh People’s Hospital Affiliated to Shanghai University of Traditional Chinese Medicine, Shanghai 200137, China; zhai_xx@126.com

**Keywords:** *Daphne*, diterpenoid, bioactivities

## Abstract

There are abundant natural diterpenoids in the plants of the genus *Daphne* from the Thymelaeaceae family, featuring a 5/7/6-tricyclic ring system and usually with an orthoester group. So far, a total of 135 diterpenoids has been isolated from the species of the genus *Daphne*, which could be further classified into three main types according to the substitution pattern of ring A and oxygen-containing functions at ring B. A variety of studies have demonstrated that these compounds exert a wide range of bioactivities both in vitro and in vivo including anticancer, anti-inflammatory, anti-HIV, antifertility, neurotrophic, and cholesterol-lowering effects, which is reviewed herein. Meanwhile, the fascinating structure–activity relationship is also concluded in this review in the hope of providing an easy access to available information for the synthesis and optimization of efficient drugs.

## 1. Introduction

The genus *Daphne* Linn., with its ca. 95 species, is the most diverse genus in the Thymelaeaceae family [1]. Some of the species from the genus *Daphne* have been applied for a long history in traditional treatments for aches, rheumatism, inflammation, and abortion in Asia, Africa, and Europe [2]. Yet, none of the principles of these bioactivities had been identified until daphnetoxin was isolated as a major toxic principle from commercial “mezeron” bark which was made from *Daphne mezereum* L. and other *Daphne* species in 1970. Subsequently, an increasing number of diterpenoids have been discovered from *Daphne* species.

The diterpenoids are believed to be representative components of the genus *Daphne*, and the genus *Daphne* itself also acts as an important role in the discovery of phytochemical and bioactive properties of diterpenoids. The archetype of one class, daphnetoxin, was first isolated from *D. mezereum,* which was named after the genus *Daphne*. Then, daphnetoxin and its analogues have been collectively known as the daphnetoxin class. Similarly, genkwanine A from *D. genkwa* is the archetypical diterpenoid of genkwanines.

The diterpenoids from the *Daphne* genus also contribute to the pharmacological study of diterpenoids and have been demonstrated to possess a variety of important biological activities including anticancer, anti-inflammatory, anti-HIV, antifertility, neurotrophic, and cholesterol-lowering effects [3], and some of them are undoubtedly efficient agents which have the potential to be developed as new drugs, such as yuanhuacine and genkwadaphnin.

The current review provides a comprehensive coverage of all natural diterpenoids in the genus *Daphne*. The occurrence and distribution of these diterpenoids are also discussed thoroughly, including the source species of every diterpenoid listed in a chronological order of discovery and the parts of plants which they were isolated from. Besides, detailed information on every class of diterpenoids is provided in this review. When the adequate information is given, the structure–activity relationship (SAR) is discussed.

## 2. Classification, Structures and Origins 

**Figure 1:**
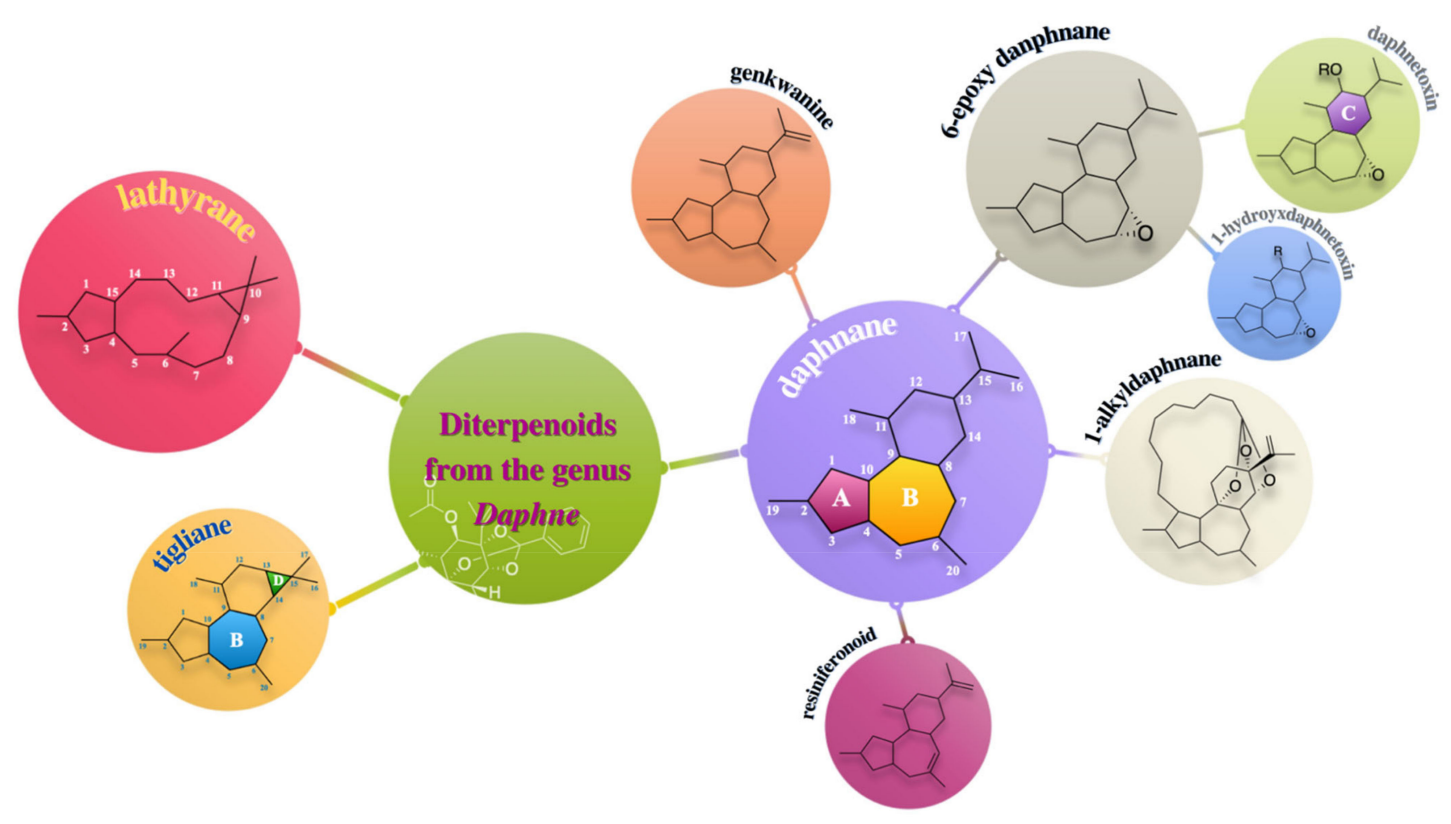
Classification and skeletons of diterpenoids from the genus *Daphne*.

So far, there are three types of diterpenoids, daphnane, tigliane and lathyrane, isolated from species of the genus *Daphne* in total. Most of the natural diterpenoids occurring in the genus *Daphne* belong to the daphnane type featuring a 5/7/6-tricyclic ring system with polyhydroxyl groups at C-3, C-4, C-5, C-9, C-13, C-14, or C-20 and an orthoester function located at ring C, which could be further categorized into four major classes:

- 6-epoxy daphnane diterpenoids (**1**–**83**), sharing the characteristic of an epoxy ring at C-6 and C-7;
- genkwanines (**84**–**95**), possessing a saturated ring A and a 6,7-dihydroxyl group in the ring B;
- resiniferonoids (**96**–**97**), which could be viewed as 5-deoxy-6,7-double bond daphnetoxin derivatives;
- 1-alkyldaphnanes (**98**–**107**), featuring a saturated ring A and a macrocyclic bridge connecting C-1 in the ring A and the end of the aliphatic orthoester group (Figure 1).

For the 6-epoxy daphnane diterpenoids, they could be further subdivided into 12-hydroxydaphnetoxins (**1**–**54**) and daphnetoxins (**55**–**83**) according to the possession of an oxygen group at C-12 in the ring C [3].

Nonetheless, it is worth noticing that the existing classification system could not apply to some daphnane diterpenoids (**108**–**123**). As for compounds **108**–**112,** the skeleton resembles the genkwanine one but possesses a 3-ketone group, a C-12 oxygenated substituents, and an additional 1,2-double bond, which makes it not accord with the definition of genkwanine. What attracts more attention is the unique 4,7 or 4,6-ether structure of neogenkwanine A-I and daphneodorin H (**113**–**123**), which is believed to never be found in any other daphnane diterpenoid [4].

Besides daphnane diterpenoids, tigliane diterpenoids (**124**–**133**) in *Daphne* species, instead of a caged 9,13,14-orthoester, distinguish themselves with a cyclopropane ring D, which is regarded to be closely related to the daphnane one [5,6]. Meanwhile, two lathyranes (**134** and **135**) were also reported to be isolated from *Daphne genkwa*, featuring a characteristic 5/11/3-membered ring system.

**Scheme 1:**
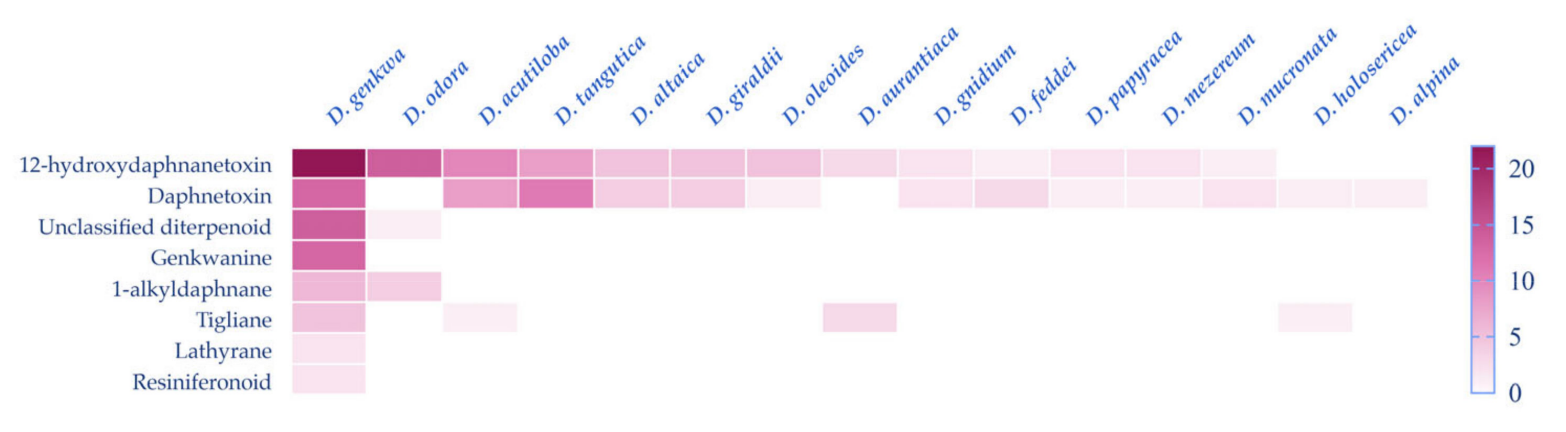
Distribution of diterpenoids in the species of the genus *Daphne*.

Although a large amount of diterpenoids were isolated and identified, they were reported to occur mainly in Thymelaeaceae and Euphorbiaceae and to mainly distribute in the genus *Daphne*, *Wikstroemia* and *Stellera* in the Thymelaeaceae family, as well as *Excoecaria* and *Euphorbia* in the Euphorbiaceae family [3]. In terms of the genus *Daphne*, diterpenoids are reported to be obtained from fifteen *Daphne* species including *D. acutiloba*, *D. altaica*, *D. alpina*, *D. aurantiaca*, *D. feddei*, *D. genkwa*, *D. giraldii*, *D. gnidium*, *D. holosericea*, *D. mezereum*, *D. mucronata*, *D. odora*, *D. oleoides*, *D. papyracea,* and *D. tangutica* (Scheme 1).

Diterpenes are abundant in several *Daphne* species including *D. odora*, *D. acutiloba*, and *D. tangutica*, especially in *D. genkwa.* It is intuitively demonstrated that diterpenoids from the *Daphne* genera are mainly of the 6-epoxy daphnane-type. In *D. genkwa*, diterpenoids of every class discussed in this review have been isolated, which may suggest a variety of diterpenoids in *D. genkwa*. Interestingly, it is also observed that the quantity of 12-hydrodaphnetoxins is much larger than that of daphnetoxins in *D. odora* while the amounts of these two types are nearly equivalent in other species, and the biogenetic mechanism behind this remains unclear.

### 2.1. Daphnane-Type Diterpenoids

#### 2.1.1. 6-Epoxy Daphnane Diterpenoids

The diterpenoids from this class share the common features of a 6α,7α-epoxy and 4β,5β-dihydroxy in the seven-membered ring B. The 6-epoxy daphnane diterpenoids could be further divided into two classes, 12-hydroxydaphnetoxins (**1**–**54**) and daphnetoxins (**55**–**83**), based on the oxygenated substituent at C-12 in the ring C.

##### 12-Hydroxydaphnetoxins

**Figure 2:**
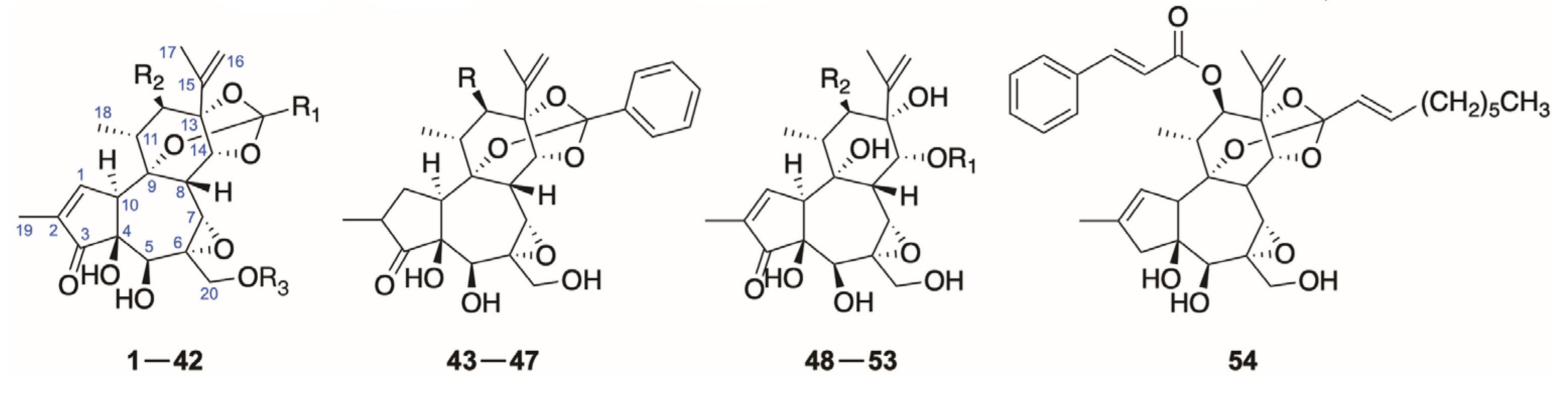
Structures of 12-hydroxydaphnetoxins (**1**–**54**).

Compared to daphnetoxins, 12-hydroxydaphnetoxins have an additional oxygen group at C-12. There are more 12-hydroxydaphnanetoxins than daphnetoxins existing in the species from the *Daphne* genera, including 1,2-dihydro derivatives (**43**–**47**), 3-deoxy derivative (**54**), and the ones with a 5,20-acetonide (**27** and **28**). The archetypal compound of 12-hydoxydaphnanetoxins is 12-hydroxydaphnetoxin (**39**), which was first found as a degradation product in *Lasiosiphon Burchellii* Meisn. [7] (Figure 2).

The species from the Thymelaeaceae are rich sources of 12-hydroxydaphnanetoxins, especially the genus *Daphne*. Compounds of this class were mainly isolated from *D. genkwa*, *D. tangutica,* and *D. acutiloba* and these diterpenoids were abundant in the flower buds of *D. genkwa*, stems, roots, and especially bark of the plants (Table 1).

##### Daphnetoxins

**Figure 3:**
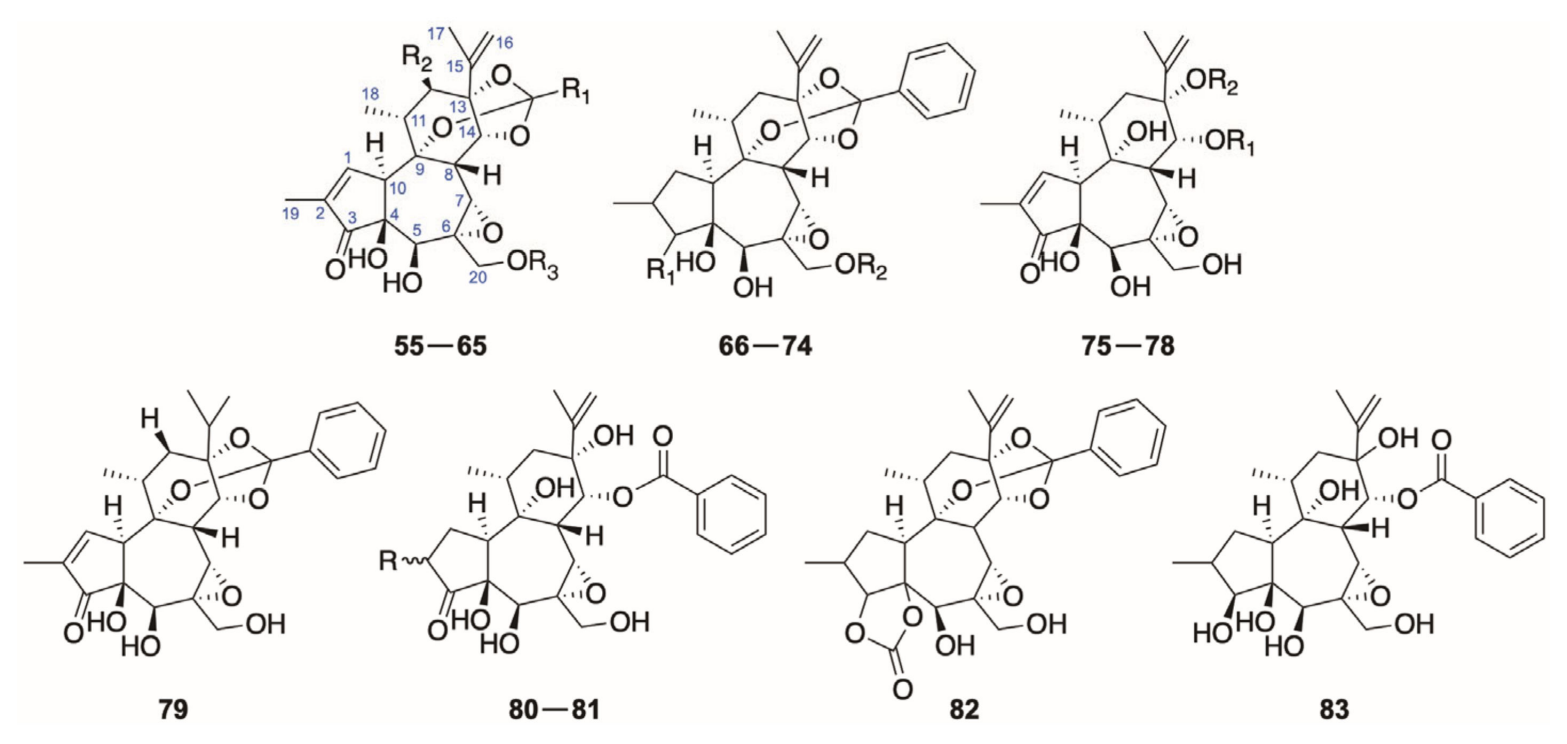
Structures of daphnetoxins (**55**–**83**).

There are less compounds from the genus *Daphne* belong to daphnetoxins than 12-hydrodaphnetoxins including a 15,16-dihyrdo derivative tanguticahine (**79**). The archetype of this class is daphnetoxin (**14**), which was separated from the bark of *D. mezereum* for the first time [61] and later proved to exist also in *D. papyracea* [13], *D. giraldii* [52], *D. tangutica* [25], and *D. acutiloba* [8] in chronological order (Figure 3 and Table 2).

#### 2.1.2. Genkwanines

**Figure 4:**
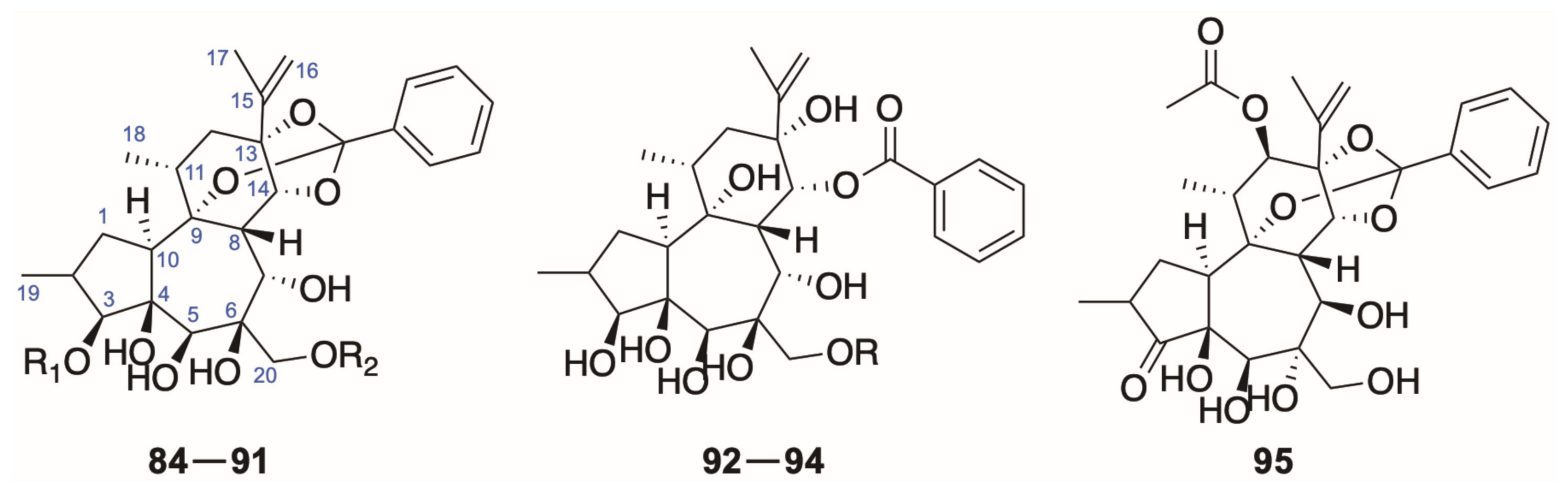
Structures of genkwanines (**84**–**95**).

The highly oxygenated diterpenoids genkwanines has a 6,7-dihydroxyl in the seven-membered ring B instead of a 6α,7α-epoxy in 6-epoxy daphnane diterpenoids or a 6,7-double bond possessed by resiniferonoids and also a saturated ring A, which could be almost viewed as 1,2-dihydro-3-hydroxy-daphnetoxin derivatives. The archetypal diterpenoid of this class is genkwanine A (**84**). Among these genkwanines, only genkwanine L (**95**) possesses a ketone function at C-3 and an oxygen-containing function at C-12 (Figure 4).

Up to now, all compounds of this class were reported to occur in *D. genkwa*, and these compounds were isolated only from the flower (mostly buds) of *D. genkwa* [43,54,55,57,66,73] (Table 3).

#### 2.1.3. Resiniferonoids

**Figure 5:**
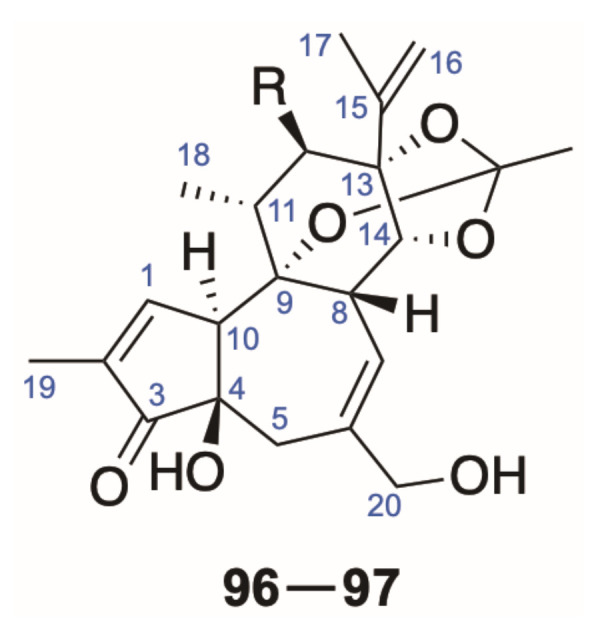
Skeleton of resiniferonoids (**96**–**97**).

The resiniferonoids are a group of 5-deoxy-6,7-double bond daphnetoxin derivatives, in which the A/B ring system possesses the pattern of phorbol. One of the most representative resiniferonoids, resiniferatoxin (RTX), was isolated from the dried latex of *E. resinifera,* showing significant transient receptor potential vanilloid 1 (TRVP1) activating activity and strong irritant effect [3,5], and compounds discovered in this class so far contain the same skeleton as RTX (Figure 5).

Resiniferonoids have a quite narrow distribution, mostly limited to several *Euphorbia* genus in the Euphorbiaceae [3,5]. Two novel resiniferonoids daphneresiniferins A and B (**96** and **97**) obtained from the *Daphne* species [43] are practically identical to each other, except for the variation of oxygenated functions at C-12, which is quite rare and noteworthy (Table 4).

#### 2.1.4. 1-Alkyldaphnanes

**Figure 6:**
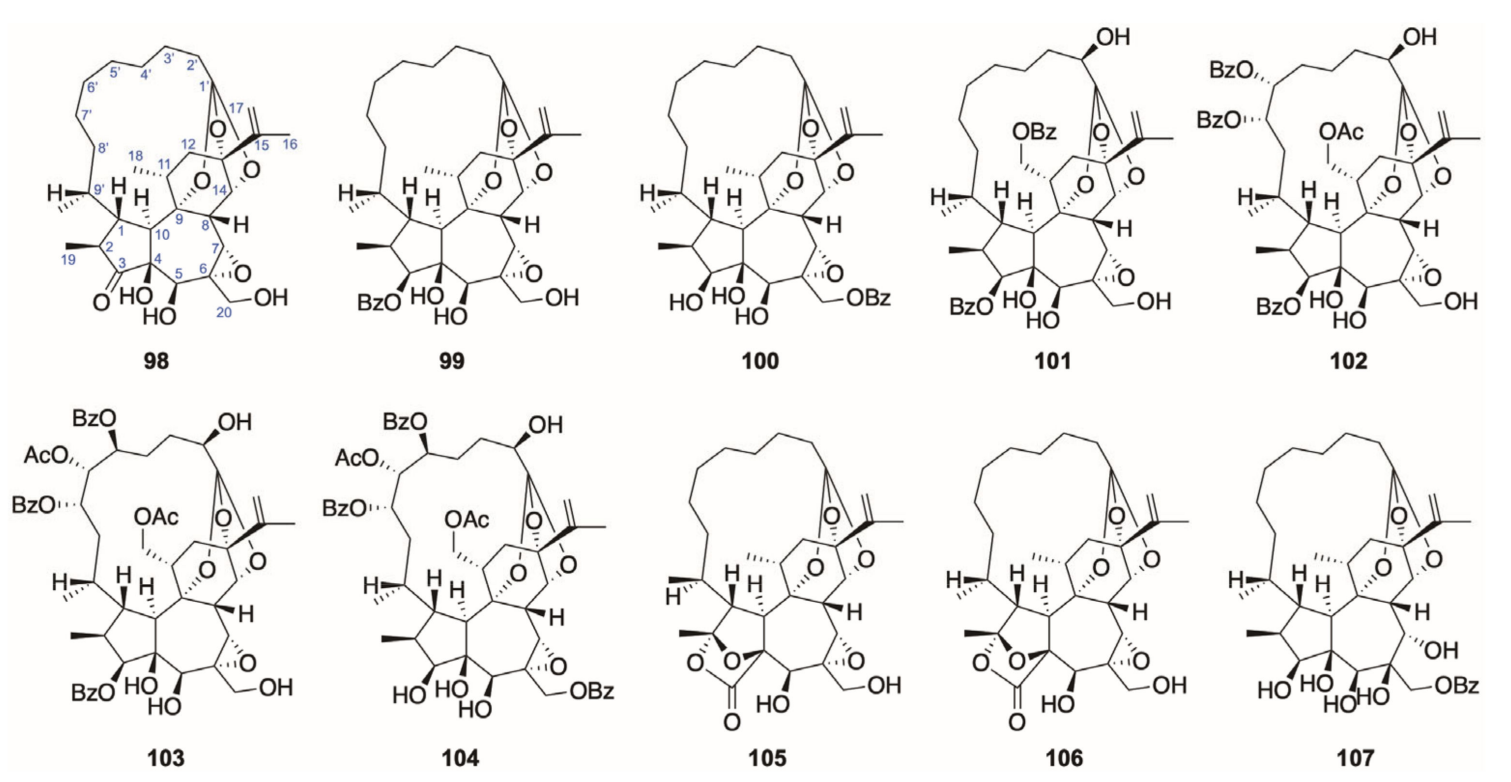
Structures of 1-alkyldaphnanes (**98**–**107**).

The 1-alkyldaphnanes (**98**–**107**) have an obvious feature of bridging between the C-1′ at the end of 9,13,14-orthoester group and C-1 in the ring A with a C-C bond. Meanwhile, the ring A of 1-alkyldaphnanes is usually saturated. The 1-alkyldaphnanes also has two subdivisions, 3-hydroxy and 3-acyloxy classes, depending on the oxidation degree of C-3 (Figure 6).

The archetype of this category is gnidimacrin (**101**), which was obtained from *Gnidia subcordata* originally [75] along with its 20-palmitate, and it was also isolated from the *Daphne* plants. The 1-alkyldaphnanes (**98**–**107**) tend to exist mainly in the buds of *D. genkwa* [14,44] and branches of *D. odora* [76]. As compared to *Daphne* genera, there appeared to be more 1-alkyldaphnane diterpenoids in the leaves and roots of the plants from the *Stellera* [77,78,79] and *Wikstroemia* genera [80,81,82] (Table 5).

#### 2.1.5. Other Daphnane-Type Diterpenoids

**Figure 7:**
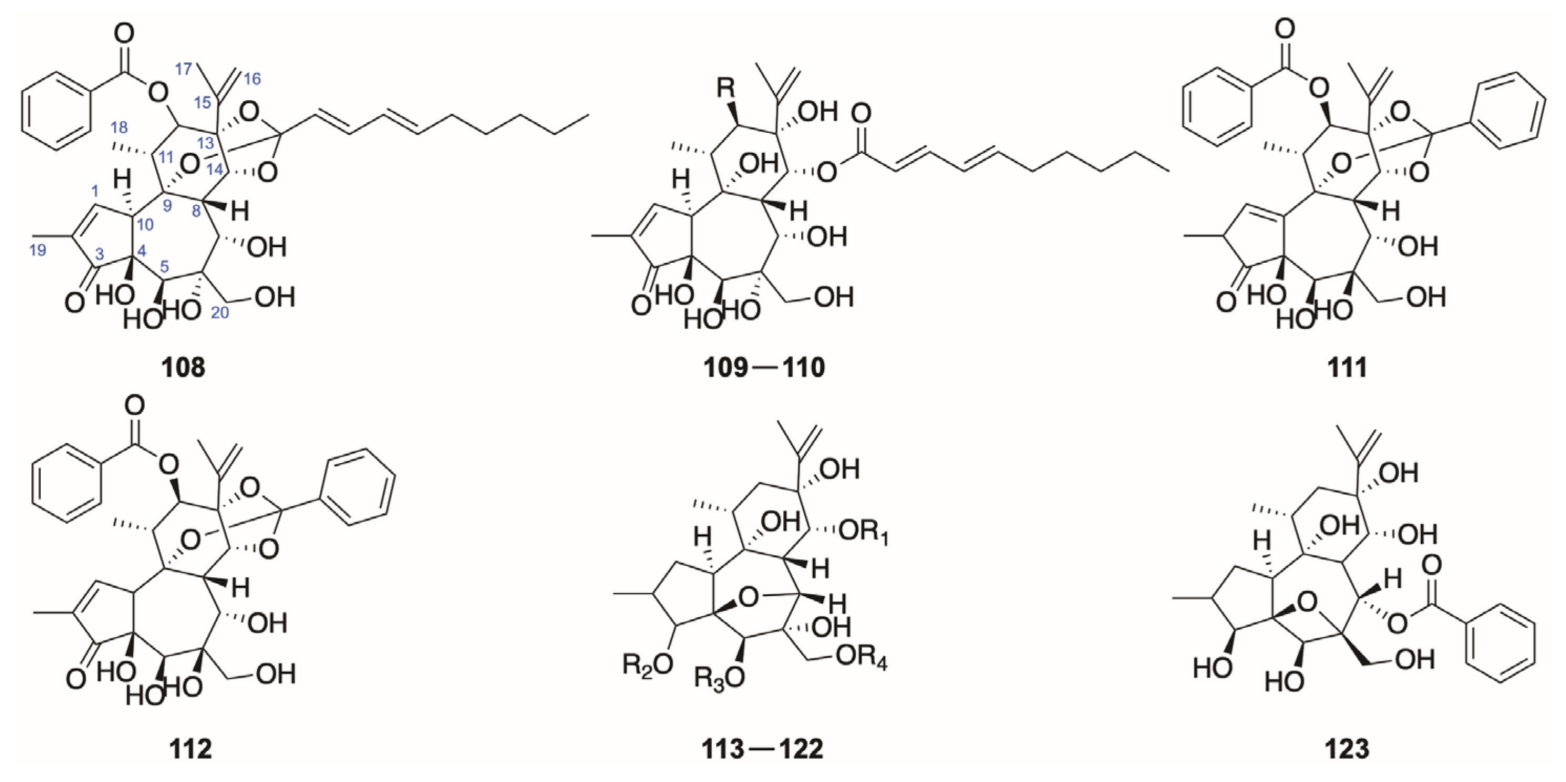
Structures of other daphnane diterpenoids from the genus *Daphne* (**108**–**123**).

Some daphnane diterpenoids (**108**–**123**) remain uncategorized into four classes mentioned above, which could be divided into two major types. The skeleton of daphgenkin A–C (**109**–**111**) and yuanhuaoate B (**112**) resembles the one of genkwanines (Figure 4), but with the variations of a 3-ketone function, an oxygenated group at C-12, and a 1,2-double bond (Figure 7).

Genkwanine L (**95**, Figure 4), mentioned in Section 2.1.2, is classified in this review as a genkwanine diterpenoid according to earlier reviews [3,83]. It was regarded as an exception in genkwanines by some researchers [3] at that time with the possession of a 3-ketone and a 12-acetoxy, which is noteworthy. Neogenkwanine A-H, genkwanine G, daphneodorin H, and genkwanine VIII (**113**–**122**) feature a 4,7-epoxy-bridged structure (a 4,6-epoxy-bridged one in neogenkwanine I) in ring B, which is very rare in daphnane diterpenoids, yet there is no appropriate category for these diterpenoids. These diterpenoids (**108**–**123**) were obtained from the buds of *D. genkwa* with only the exception of daphneodorin H (**113**) isolated from the leaves and branches of *D. odora* (Table 6).

### 2.2. Tigliane-Type Diterpenoids

**Figure 8:**
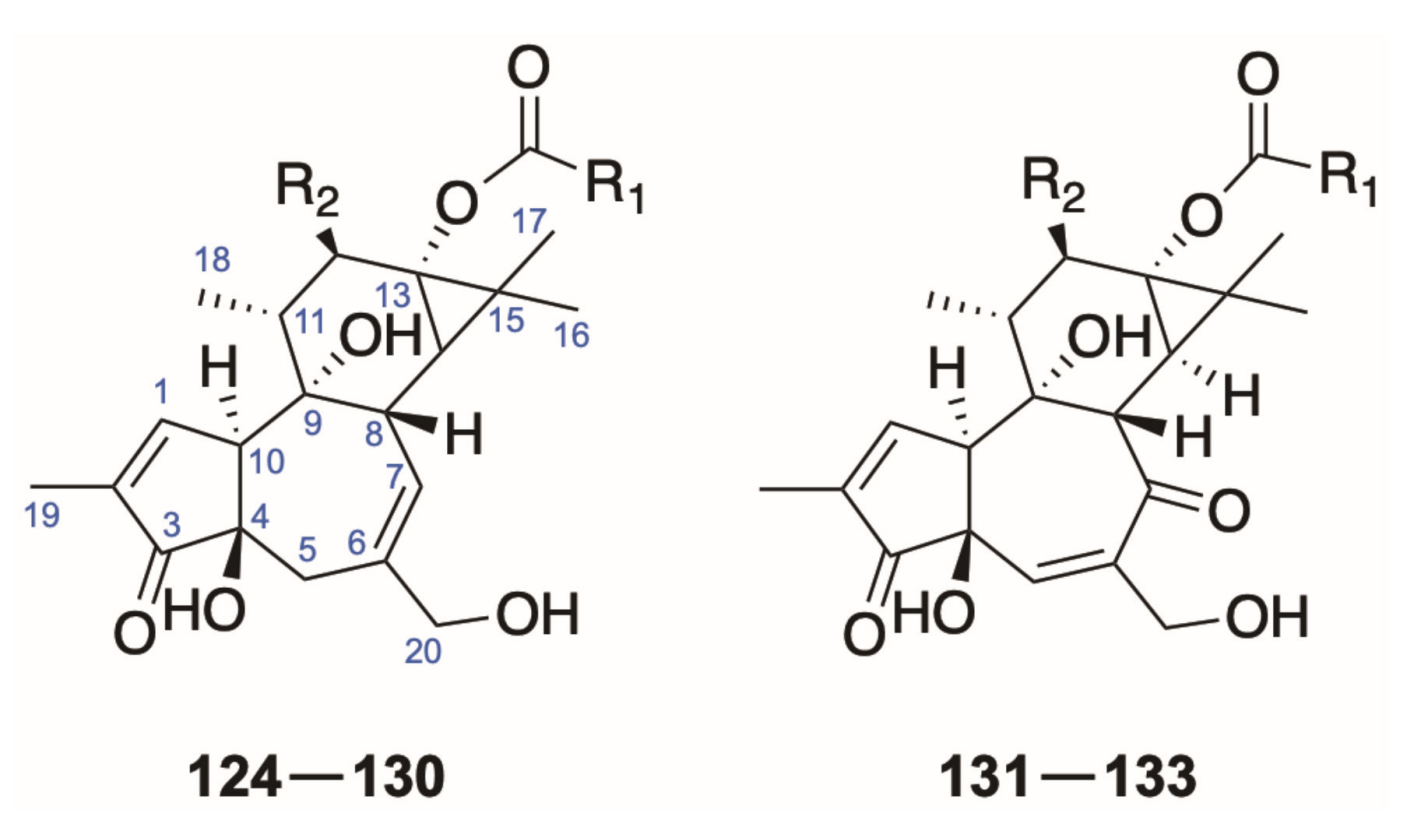
Structures of tigliane-type diterpenoids (**124**–**133**).

The tigliane tetracyclic diterpenoids share a closely related skeleton with daphnane ones with a highly substituted 13,14-cyclopropane ring D [6], presenting potent irritant and cocarcinogenic activities [86]. One representative and parent diterpene in this class is phorbol isolated from *Croton tiglium* [86]. The tigliane diterpenes widely distribute in many species from the genus *Daphne* [20,65,87], *Pimelea* [88], and *Stellera* [89] in the Thymelaeaceae fam, as well as *Euphorbia* [90,91] and *Jatropha* [86] in the Euphorbiaceae family. As compared to the phorbol esters (**124**–**130**), there are structural variations of a 7-ketone function and a 5,6-double bond in the ring B in the others (**131**–**133**, Figure 8 and Table 7).

### 2.3. Lathyrane-Type Diterpenoids

**Figure 9:**
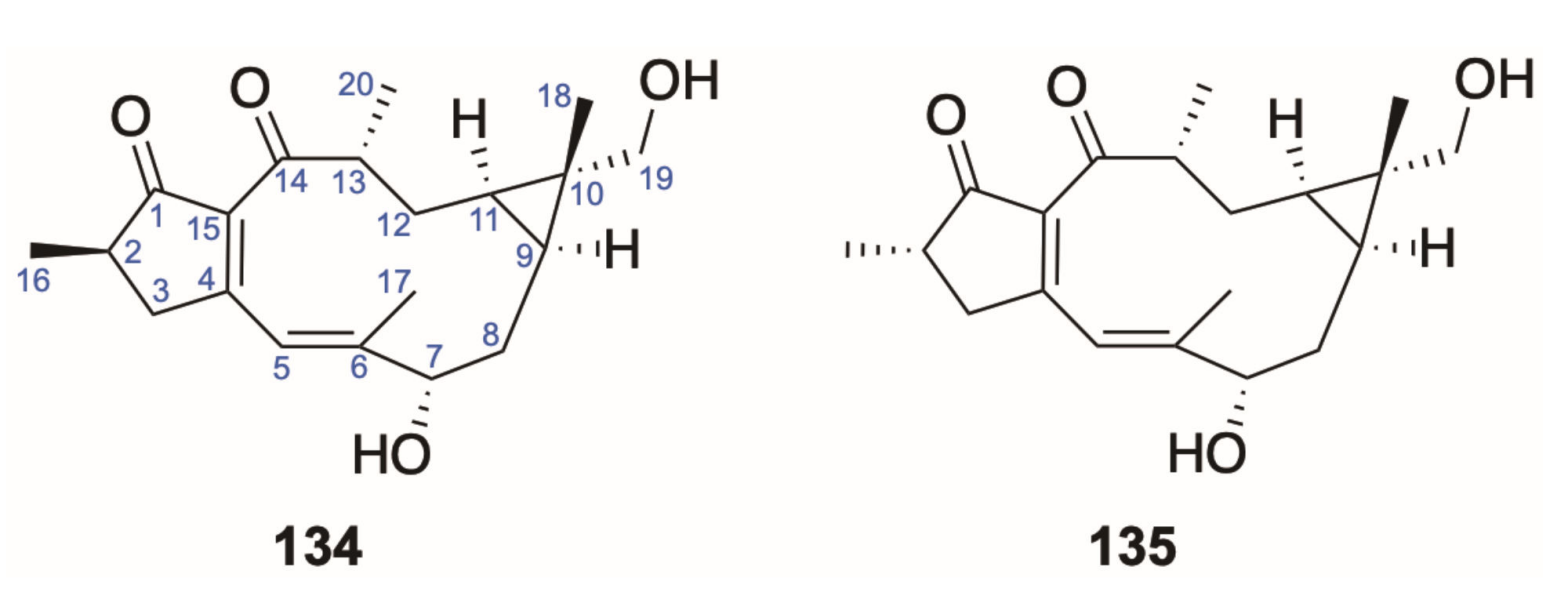
Structures of lathyrane-type diterpenoids (**134**–**135**).

Lathyranes, named after *Euphorbia lathyris*, were more often found in species from the Euphorbiaceae other than those in the Thymelaeaceae, especially in the *Euphorbia* species [86,93]. The archetypal compound of lathyrane is 17-hydrojolkinol obtained as a derivative product of ‘ester 7′ from the seeds of *E. lathyris* [94]. As for the skeleton with an additional 4,15-bond possessed by **134** and **135**, it was believed to be a precursor for crotofolin from *Croton corlifolious* [86] (Figure 9).

So far, these are the only two lathyrane diterpenoids reported to be isolated from the *Daphne* species [66] (Table 8).

## 3. Biological Activities

Several *Daphne* plants have been used as traditional medicines for the treatment of cancer, inflammation, and rheumatism in Asia, North Africa, and Europe, and some of these plants were also regarded as virulent poisons. The flower bud of *D. genkwa*, a Chinese traditional medicine, has been used as a diuretic, antitussive, and pesticide, and one of its synonyms is “yu-du”, which means “a fish poison” [1,2,95].

However, the poisonous principles of any *Daphne* species have never been identified until daphnetoxin (**56**) was isolated from commercial “mezeron” bark (*D. mezereum*, *D. laureola*, and *D. gnidium*) in 1970 and identified as a major toxic component possessing both a similar skeleton and similar sites of functions as phorbol, which was a known toxic principle in *Croton* species [61,96] at that time. Subsequently, mezerein (**24**) with the same skeleton as daphnetoxin (**56**) was identified as another toxic principle of *D. mezereum* [33].

With a considerable number of modern pharmacological and chemical studies, it was demonstrated that diterpenoids from the *Daphne* genus possess a wide range of pharmacological activities including anticancer, anti-inflammatory, anti-HIV, cholesterol-lowering, neurotrophic, antifertility, skin irritant, nematicidal, and pesticidal activities. Among these biological activities, the anticancer ones have received the most attention since there remains a need for both efficient and safe anticancer drugs with novel structures.

### 3.1. Anticancer Activity

**Scheme 2:**
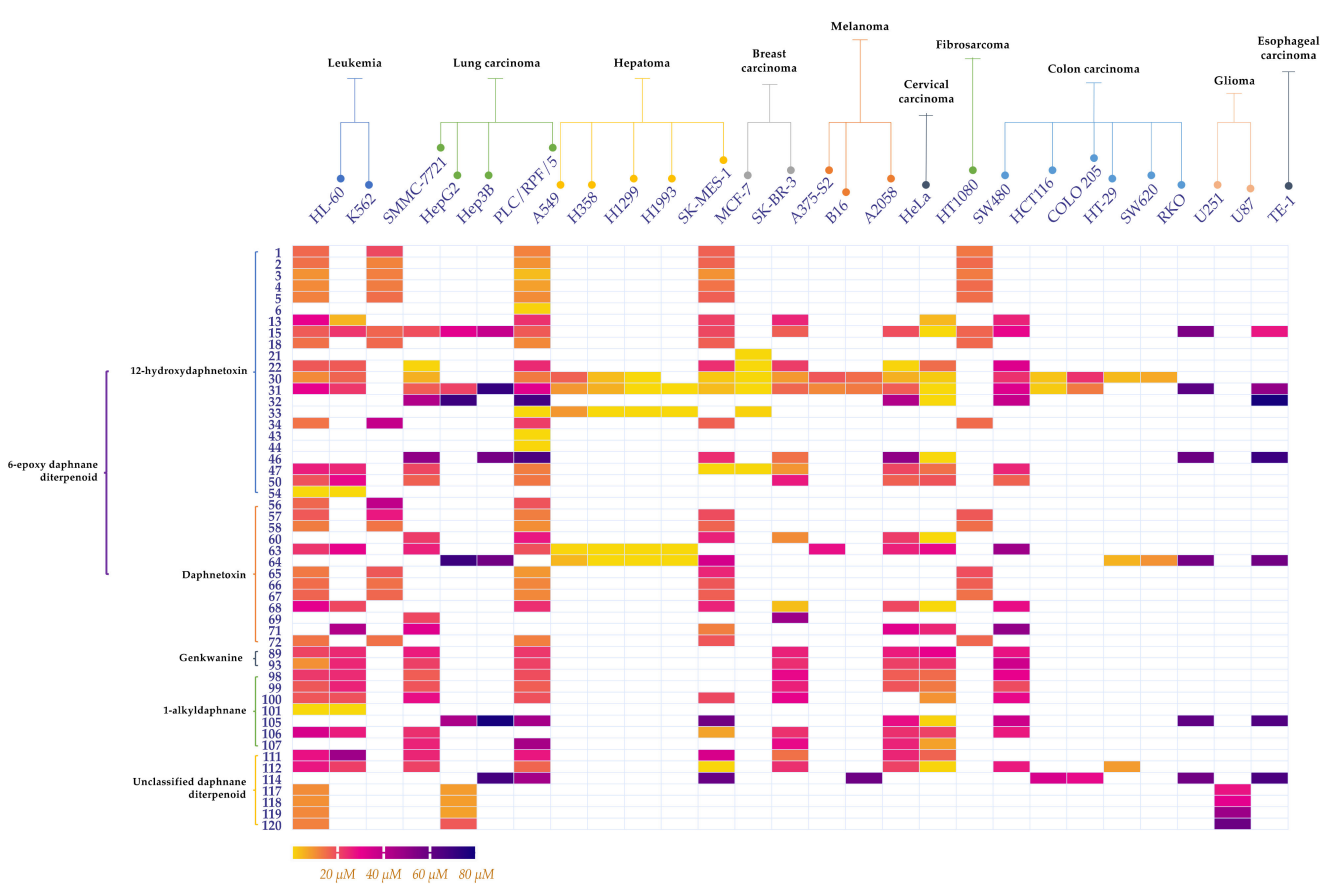
Heat map of the IC_50_ values (μM) of cytotoxicity of some diterpenoids in various carcinoma cell lines in vitro.

Previous studies have shown that diterpenoids from *Daphne* species exhibit potent anticancer activities against various types of cancers both in vitro and in vivo [2,83,97]. As for anticancer bioactivities in vitro, more than a half of the diterpenoids have been testified to exhibit cytotoxicity with IC_50_ values ranging from 10^−6^–98.46 μM (Table S1) against various cell lines, all of which belong to the daphnane type (Scheme 2).

The cytotoxicity of 51 diterpenoids against certain cell lines are intuitively shown in Scheme 2. As for the cell lines sensitive to very few diterpenoids and the diterpenoids only cytotoxic to a very limited number of cell lines, they will be additionally discussed below.

#### 3.1.1. Structure–Activity Relationship (SAR)

**Figure 10:**
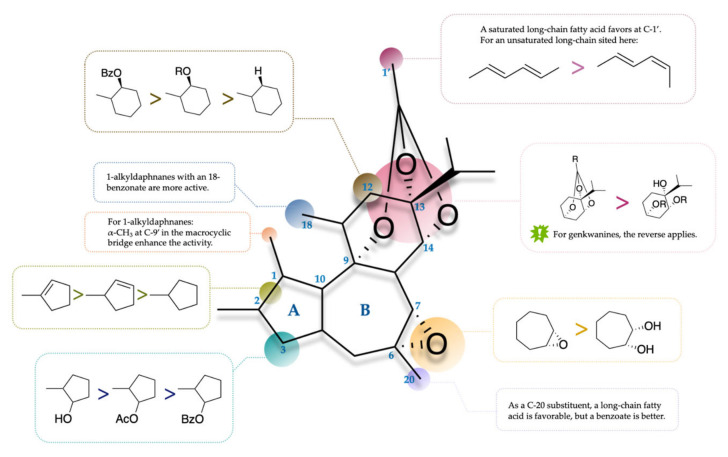
SAR of diterpenoids as antineoplastics.

For an intuitive demonstration of the structure–anticancer activity relationship of diterpenoids from the genus *Daphne*, the structure and functions requirements are illustrated with a daphnane diterpenoid skeleton (Figure 10).

##### SAR in the Ring A

It is generally accepted that the 1,2-double bond could be a possible alkylating functionality in a diterpenoid and thus the reduction of it might make it a less active compound [98]. Genkwadaphnin (**15**), yuanhuatine (**47**), and yuanhuaoate B (**112**) share the similar structure with the only exception of an unsaturated bond in the ring A, and yuanhuatine (**47**) among them is the least active anticancer compound [8,14]. The site of an unsaturated carbon bond in the ring A would also have certain affect. For genkwadane A (**111**) and yuanhuaoate B (**112**), it appears that the change from the 1,10- to 1,2-double bond enhances the anticancer activity [14].

In the ring A, the variation of the oxygen-containing function at C-3 influences the anticancer activity. 12-*O*-benzoyl-3,5-hydroxy-6,7-epoxy-resiniferonol-9,13,14-orthobenzoate, which is a 3-hydroxyl derivative of genkwadaphnin (**15**), showed a promising cytotoxicity against seven carcinoma cell lines, much better than genkwadaphnin (**15**) itself, indicating the possibility that the hydronation of a 3-ketone favors a bioactivity [99]. However, genkwanine A (**84**) with a 3-hydroxyl was basically inactive against A549 cells, while its 3-acylated analogues (**85**–**87**) all possessed certain cytotoxicity with the IC_50_ values in the range of 0.79–8.70 μM [73], indicating that a 3-acyloxy function enhances the anticancer activity of diterpenoids with a more saturated skeleton. Genkwanine D (**87**) was the most potent one with a 3-benzoate group at the IC_50_ values of 0.79–8.00 μM [73] suggesting that a benzoate group at C-3 might favor the antineoplastic effect.

##### SAR in the Ring B

The 6α,7α-epoxy is a characteristic structure in 6-epoxy and 1-alkyldaphnane diterpenoids, and it was reported to help enhance the anticancer activity. Yuanhuacine (**30**), yuanhuadine (**31**), yuanhuafine (**32**), and yuanhuapine (**46**) generally showed stronger cytotoxicity against A549 cell line than genkwanine A-L (**84**–**95**) possessing a 6α,7α-dihydroxyl in the same assay [73]. Additionally, genkwadane B (**100**) possessed a stronger activity in seven cell lines than genkwadane C (**107**) [14], suggesting that the opening of 6α,7α-epoxy has a negative effect on antineoplastic activity as well.

The 20-acyloxy group in the ring B might have an important role in affecting the anticancer activity in diterpenoids. One type of 20-acylocy groups that could favor the bioactivity is a 20-palmitate ester. Yuanhuacine (**30**) showed no inhibitory activity against P-388 in vivo, while its 20-palmitate derivative gnidilatidin 20-palmitate (**19**) exhibited a strong inhibitory activity at dosages of 0.5–2.0 mg/kg/d, and the 20-plamitate derivative of gnidilatin was also observed to be more active than gnidilatin itself against P-388 [100]. This fact might also suggest the positive impact of a long-chain fatty ester on determining the anticancer activity.

Genkwanine M (**68**) with a 20-benzoate was demonstrated to inhibit various cell lines more significantly, especially HL-60 leukemia cells, A375-S2 melanoma cells, and HT-1080 fibrosarcoma cells, than orthobenzoate 2 (**71**) [14]. Similarly, genkwanine H (**91**) possessing a 20-benzoate exhibit much stronger cytotoxicity against P388 and A549 (IC_50_: 13.0 and 1.60 μM) than its analogues [58], indicating that an aromatic acyl group at C-20 might also enhance the anticancer activity.

##### SAR in the Ring C

The caged 9,13,14-orthoester is another characteristic structure in the daphnane-type diterpenoids in general. Genkwanines J–K (**93**–**94**) with a 9,13,14-orthoester were more cytotoxic (IC_50_: 4.20–42.0 μM) than genkwanine F–G (**89**–**90**, (IC_50_: 24.0–57.0 μM) in P-388 and A549 cells [73].

Interestingly, for a more statured skeleton, for instance, genkwanine, the caged 9,13,14-orthoester seems to reversely have a negative impact on anticancer activity. Genkwanine J (**92**) showed potent inhibition against P388 and A549 (IC_50_: 4.2 and 25.0 μM), while genkwanine F (**84**) were moderately active with the IC_50_ values of 39.0–24.0 μM [73]. More studies have further shown a more significant anticancer activity of genkwanine J (**92**) compared to genkwanine F (**84**) in different cell lines [14].

Evidence showed that the presence of a long-chain fatty could enhance the inhibitory activity against carcinoma cells. Yuanhuacine (**30**) and yuanhuadine (**31**) showed promising inhibition against HepG2 cell line with the IC_50_ values in the range of 5.56 to 17.06 μM, while yuanhuafine (**32**) showed more limited activity at the IC_50_ value of 42.37 μM [14].

Whether this long chain at the end of the 9,13,14 orthoester is statured or not could influence the antineoplastic activity as well. Gnidilatin was a potent antileukemia compound against P-388 in vivo; meanwhile, yuanhuacine (**31**) was inactive with the introduction of 2′,3′- and 4′,5′-double bonds [100]. Yuanhuagine (**33**) with one more unsaturated bond in the long-chain substituent in orthoester group presents weaker bioactivity (IC_50_: 809.4 nM) than yuanhuadine (**31**) and isoyuanhuadine (**22**, IC_50_: 61.6 and 83.7 nM) in SK-BR-3 [30], which indicates that the introduction of unsaturated bond in the 9,13,14 orthoester structure is unfavorable.

As for the unsaturated long-chain at C-1′, the stereochemistry matters. Yuanhuacine (**30**) and yuanhuadine (**31**) possessing a 2′*E*,4′*E*-double bond could both inhibit SK-BR-3 proliferation more significantly (IC_50_: 172.6 and 61.6 nM) than their conformational isomers, respectively (IC_50_: 217.1 and 83.7 nM) [30].

The absence of a 12-acyl group generally causes the reduction of anticancer bioactivity. Mezerein (**24**), a major toxic principle of *D. mezereum*, showed significant inhibitory activity in vivo against P-388 and L-1210 leukemia cell lines at dosage of 50 μg/kg in mice [34]. Gnidicin (**16**), gnididin (**18**), and gniditrin (**20**) with the closely related skeleton isolated from *Gnidia lamprantha* Gilg were also proved to be substantial antileukemia agents. Huratoxin (**59**) and simplexin (**60**) exhibited similar inhibitory activities against L1210 and K562 in vitro, but less active than an esterified derivative subtoxin [77]. By comparison, 12-hydroxydaphnetoxin (**39**) bearing a hydroxyl at C-12 in the ring C showed no antileukemia activity and esterification of it could establish the bioactivity [101]. Genkwadaphnin (**15**), which is in essence the benzoyl derivative of 12-hydroxydaphnin (**39**), was found to exert both in vitro and in vivo antileukemia activity against P-388 cells [16]. Although all the above together suggests that an acyl function at C-12 might be a prerequisite for the in vivo antileukemia bioactivity, this does not represent that a 12-acyloxyl group is necessary for antileukemia activities regarding the fact that some daphnetoxins were reported to show a quite promising inhibition against a variety of cell lines including HL-60 and K562 as well (Scheme 2).

The type of ester group at C-12 may also have an impact on the antileukemia activity. Yuanhuacine (**30**) showed stronger cytotoxicity against HL-60 and K562 cells (IC_50_: 26.81 and 16.08 μM) than yuanhuadine (**31**, IC_50_: 30.05 and 22.16 μM). Genkwadaphnin (**15**), with a 12-benzoate function, was also more active in antileukemia than yuanhuafine (**32**), as the latter showed limited inhibitory activity in HL-60 and K562 cell lines [14]. Yuanhuatine (**47**) was cytotoxic against human and chronic myeloid promyelocytic leukemia cell lines at the IC_50_ values of 17.72 and 17.54 μM [14], while yuanhuapine (**46**) has not been reported to possess antileukemia property. Yuanhuacine (**30**) and isoyuanhuacine (**21**) showed moderate cytotoxicity against SK-BR-3 (IC_50_: 172.6 and 217.1 nM), while and yuanhuadine (**31**) and isoyuanhuadine (**22**) were more active (IC_50_: 61.6 and 83.7 nM) [30]. These verify that a benzoyl group at C-12 might act as an important role in affecting the antileukemia activity other than an acetoxyl one.

It is noteworthy that gnidimacrin (**101**) is one of the most potent antileukemia agents both in vitro (IC_50_ in the range of 0.16–0.28 nM) to HL-60, K562, and CCRF-CEM cells [78] and in vivo at dosages of 0.02–0.03 mg/kg/d [78,79], which is also much more active than other 1-alkyldaphnanes isolated from *Daphne* species [57]. It is generally considered that the antineoplastic activity is related to the 18-benzoyloxy substituent at C-18 [3,102].

Interestingly, for pimelotide A (**105**) and pimelotide C (**106**), it seems that the one with α-CH3 (**106**) exhibits more significant antineoplastic bioactivities against a variety of cell lines including HeLa, MCF-7, HepG2, HCT116, A549, A375-S2, U937, HL-60, and K562, which may suggest that the stereochemistry of substituents is related to the anticancer activity as well [14].

#### 3.1.2. Anticancer Activity and Involved Mechanisms

##### Leukemia

It is also revealed that genkwadaphnin (**15**) could oppose the protein and DNA synthesis of P-388 cells to exert both in vitro and in vivo antileukemia activities [103,104]. Genkwadaphnin (**15**, IC_50_: 11.8 μM) and yuanhuacine (**30**, IC_50_: 10.8 μM) were determined to suppress Bcl-2 and Bcl-X_L_ in a dose-dependent manner to induce apoptosis in human myelocytic HL-60 cells [39].

Gnidimacrin (**101**) showed a significant antiproliferative effect against K562 cell lines at the IC_50_ of 1.2 nM by activating protein kinase C (PKC) and arresting the cell cycle at G_1_ phase [79].

##### Lung Carcinoma

Several 12-hydrodaphnetoxins including yuanhuadine (**31**), yuanhuadine (**63**), yuanhualine (**64**), and yuanhuagine (**33**) exhibited stronger antiproliferative activity against A549 cells (IC_50_ values in the range of 12–53 nM) than the positive control ellipticine without displaying cytotoxicity against the human normal lung epithelial cell line MRC-5, with especially yuanhuacine (**30**, IC_50_: 12 μM) presenting the most potent bioactivity [49]. Further study has indicated that yuanhuacine (**30**) could also be anticancer bioactive against H1993 human non-small cell lung cancer (NSCLC) cells both in vitro and in vivo by modulation of the AMPK/mTOR signaling pathway [105].

Yuanhualine (**64**), yuanhuadine (**63**), and yuanhuagine (**33**) showed notable inhibitory effects in drug-resistant cell lines including gemcitabine-resistant A549, gefitinib- and erlotinib-resistant H292. Further research indicated that these diterpenoids were able to arrest cell cycle in the G_0_/G_1_ and G_2_/M phase in A549 cells by upregulating the expression of cyclin dependent kinase inhibitors P21 and P53 as well as downregulating cell-cycle regulators, for example, c-Myc and cyclin B1/cell division cycle 2 (CDC2) complex and suppress Akt/STAT/Src signaling pathway [106]. Additionally, yuanhualine (**64**) was observed to have synergistic effects with certain chemotherapeutic agents (gemcitabine, gefitinib and erlotinib) in the treatment of A549 cell line [106].

##### Hepatoma

Wu et al. evaluated the effects of genkwadaphnin (**15**) on hepatocellular carcinoma (HCC) cells both in vitro and in vivo with Hep3B and PLC/PRF/5 cell lines and BALB/c nude mice, respectively, the results showed that genkwadaphnin (**15**) suppressed growth and invasion of HCC cells both in vitro and in vivo by blocking DHCR24-mediated cholesterol biosynthesis and lipid rafts formation [44]. Evidence also showed that yuanhuacine (**30**) and genkwadaphnin (**15**) were hepatotoxic on normal human liver cells HL-7702 in a dose- and time-dependent manner; meanwhile, the change of cell morphology and increased AST and ALT were observed as well [40].

##### Breast Carcinoma

Yuanhuacine (**30**) was found to be an active inhibitor in both MCF-7 and MDA-MB-231 cell lines, and the preliminary mechanism of strong cytotoxicity of yuanhuacine (**30**) against MCF-7 was investigated further by using Western blot and flow cytometry analysis; the results suggested that yuanhuacine (**30**) induced apoptosis via the regulation of Bcl-2, Bax, and cleavage of PARP expression in MCF-7 cells [84]. Yuanhuatine (**47**) was also observed to inhibit the growth of estrogen receptor alpha (ERα)-positive cells MCF-7 (IC_50_: 0.62 μM) significantly compared to tamoxifen (IC_50_: 14.43 μM) through mitochondrial dysfunction and apoptosis in ERα-positive breast cancer cells MCF-7 caused by ERα-downregulation [107]; for ERα-negative cells MDA-MB-231, either cytotoxicity or apoptosis was observed [107].

##### Melanoma

Yuanhuacine (**30**), Yuanhuatine (**47**), and genkwanine M (**68**) isolated from the buds of *D. genkwa* displayed pronounced inhibitory bioactivity against the human melanoma cell line A375-S2 at the IC_50_ levels of 8.72, 9.31, and 3.62 μM, respectively [14]. Yuanhuacine (**30**) and yuanhuadine (**31**) were obtained from the dichloromethane fraction and showed potent cytotoxicity to melanoma B16 as well as A2058 cell lines, while genkwanine C (**86**) and genkwanine VIII (**118**) were selectively active in the A2058 cells [84]. It was verified that yuanhuacine (**30**), yuanhuadine (**63**), genkwadaphnin (**15**), genkwanine A (**84**), genkwanine F (**89**), genkwanine H (**91**), and daphneresiniferin A (**96**) and B (**97**) could inhibit the α-MSH-induced melanin production in B16 melanoma cells remarkably with the IC_50_ in the range of 0.57–9.0 μM in comparison with the positive control arbutin (IC_50_: 140 μM) and kojic acid (IC_50_: 39 μM), especially yuanhuadine (**31**, IC_50_: 0.06 μM) with the striking inhibitory activity [43]. Among these compounds, a resiniferonoid daphneresiniferin B (**97**) showed obvious cytotoxicity against B16 cells at the IC_50_ level of 6.6 μM [43], which indicated that it probably affected melanin production simply with its high cytotoxicity. Genkwadaphnin (**15**) was also investigated to exert apoptosis-triggering effect in squamous cell carcinoma (SCC) cells in a JNK-dependent manner [108].

Besides, the antiproliferative activity of the ethyl acetate and aqueous extract from the leaves of *D. gnidium* was observed in B16-F0 and B16-F10 cell lines inducing G_2_/M cell cycle arrest and the ethyl acetate extract was also capable of enhancing melanogenesis stimulation activity in a concentration-dependent manner in B16-F10 cells [109]; furthermore, the aqueous extract of *D. gnidium* exerted in vitro and in vivo antimelanoma effects on B16-F10 by activating natural killer (NK) cell and cytotoxic T lymphocyte (CTL) [110], hopefully these findings might lead to the discovery of more potential compounds affecting the melanogenesis and cell cycle of melanoma cells.

##### Fibrosarcoma

The alcohol–water extract of the aerial parts of *D. mucronata* was evaluated to possess anticancer property both in vitro and in vivo and its mechanism, similar to the natural anticancer drug Taxol, was probably related to the downregulation of human tumor necrosis factor alpha receptors (TNF-αR) [111]; the probable principle of anticancer activity, gnidilatimonoein (**54**), was isolated from *D. mucronata* afterwards and showed a strong antiproliferation effect on WEHI-164 by mediating the progress of DNA synthesis [60].

##### Colon Carcinoma

The mechanism of anticancer effect of genkwadaphnin (**15**) was revealed when it was found that it enhanced the p21 expression and simultaneously suppressed the c-Myc expression in a PRDM1-denpendent manner to arrest the cell-cycle progression in the human colon cancer SW620 cell line [112].

Yuanhuacine (**30**) and yuanhuadine (**31**) were revealed to exhibit more significant inhibitory effects on the proliferation of the COLO250 (IC_50_: 2–3 μM) than HT-29 cell line (IC_50_: 13–23 μM) [84], suggesting the selectivity of the antineoplastic activity. A further study indicated that yuanhuacine (**30**) inhibited the HCT116 cell line by upregulating p21 expression and transcription via a p53 protein independent cascade [113]. Daphgenkin A (**108**), along with yuanhuacine (**30**) and yuanhuadine (**31**) obtained from the petroleum ether extract from *D. genkwa*, showed definite cytotoxic effects on both SW620 and RKO cell lines with the respective IC_50_ value of 3.0 and 6.5 μM; then, the results of further research revealed that daphgenkin A (**108**) inhibited SW620 cell proliferation by stalling the cell cycle at G_0_/G_1_ phase, causing cell death by apoptosis as well as inducing cell cycle arrest via regulating the PI3K/Akt/mTOR signaling pathway [10].

##### Gastric Carcinoma

Yuanhuacine (**30**), yuanhuadine (**31**), yuanhuatine (**47**), genkwanine F (**89**), genkwanine N (**69**), and wikstroemia factor M_1_ (**72**) obtained from *D. genkwa* were moderately cytotoxic with IC_50_ levels in the range of 25.61 to 27.32 μM against the human gastric carcinoma MGC-803 cell line [68]. Furthermore, yuanhuacine (**30**, IC_50_: 17 μM) and yuanhuadine (**31**, IC_50_: 16 μM) were detected to oppose the proliferation of human gastric adenocarcinoma AGS cell lines, along with genkwanine VIII (**118**, IC_50_: 12 μM) [84].

##### Others

Yuanhuacine (**30**) and yuanhuajine (**7**) presented more obvious inhibitory activity against DNA topoisomerase I (DNA topo I) at the IC_50_ levels of 40.0 and 38.3 μM than a known topo I inhibitor hydroxycamptothecin (hCPT, IC_50_: 48.0 μM), and further study with the prepared derivatives suggested that less electron-withdrawing groups at C-5, C-12, and C-20 facilitated the combination between the compounds and DNA topo I [47].

Yuanhuacine (**30**) was also reported to be cytotoxic against two bladder cancer cell lines UMUC3 (IC_50_: 1.89 μM) and T24T (IC_50_: 1.83 μM), and it functioned in T24T cells by inducing a G_2_/M phase arrest significantly via modulation of Sp1 protein expression [113].

The human lymphoma cell line U937 has been reported to be moderately suppressed by daphnane-type diterpene ester-7 (**50**), yuanhuacine (**30**), and yuanhuatine (**47**) with the IC_50_ levels of 11.62–12.35 μM [14], while gnidilatimonoein (**54**) showed a stronger cytotoxicity (IC_50_: 1 μM) [59].

The chloroform extract from *D. altaica* was also found to significantly suppress the proliferation of esophageal squamous carcinoma Eca-109 cell line at the IC_50_ level of 10.6 μM and in a dose-dependent manner [114], and the ethyl acetate extract of *D. altaica* was reported to function by inducing apoptosis and cell cycle arrest in the S phase in the Eca-109 cell line via the PPARγ-mediated pathway [115]. However, whether its anticancer effect is related to diterpenes or not requires detailed studies.

### 3.2. Anti-HIV Activity

Both daphnane- and tigliane-type diterpenoids from *Daphne* species were demonstrated to possess an anti-HIV activity even stronger than some anti-HIV agents such as 3′-azido-3′-deoxythymidine (AZT), and structure-activity relationship in anti-HIV bioactivity is quite similar to that in the anticancer one (Figure 10).

#### 3.2.1. Structure–Activity Relationship (SAR)

The presence of a 9,13,14-orthoester in the ring C is also favorable for anti-HIV activity as diterpenoids with an orthoester, for instance, daphneodorins D–E (**11**–**12**), presented stronger anti-HIV activity than daphneodorins F–H (**51**–**52**, **113**) without one (EC_50_ > 25 nM) [4]. Yuanhuamine A (**35**) and its isomer, isoyuanhuadine (**22**), showed promising anti-HIV activity (EC_50_ < 0.9 nM), while yuanhuaoate C (**53**) without a 9,13,14-orthoester merely exhibited moderated activity [116], suggesting that the orthoester motif enhances the anti-HIV effect.

The substituent at C-12 in the ring C acts as an important role in anti-HIV activity as well. A tigliane diterpenoid 12-*O*-benzoylphorbol-13-octanoate (**125**) from *D. aurantiaca* showed definite anti-HIV-1 activity against C8166 cell line with EC_50_ value of 0.282 nM and SI value of 65177.305, while phorbol 13-monoacetate (**130**) possessing a 12-hydroxyl showed limited activity, which suggests an acyl group at C-12 might favor anti-HIV-1 bioactivity [87].

Daphneodorins A–B (**102**–**103**) presented more potent activity in inhibiting HIV-1 replication in MT4 cell line (EC_50_: 0.16 and 0.25 nM, respectively) than daphneodorin C (EC_50_: 2.9 nM) suggesting that a 20-benzoyloxy reduces the anti-HIV activity [76].

#### 3.2.2. Anti-HIV Activity and Involved Mechanism

Prostratin (**129**) is known as a potent anti-HIV agent [117] and its mechanism is the protection of CD4+ cells by downregulation of the HIV receptor CD4 and co-receptors and the interaction with PKC to stimulate viral replication in infected cells [6]. Wikstroelide E (**98**), a HIV-latency-reversing compound that is strikingly 2500-fold more potent that prostratin (**129**), functioned by regulating various signaling pathways including the MAPK, PI3K-Akt, JAK-Stat, TNF, and NF-κB ones [116].

Acutilobins A–G (**1**–**5**, **66**–**67**, EC_50_: 0.32–1.50 nM), genkwanine N (**69**, EC_50_: 0.17 nM), genkwadaphnin (**15**, EC_50_: 1.94 nM), kirkinine (**23**, EC_50_: 5.63 nM), excoecariatoxin (**58**, EC_50_: 5.64 nM), and 14′-ethyltetrahydrohuratoxin (**65**, EC_50_: 0.52 nM) were obtained from *D. acutiloba*, all of which exhibited strong anti-HIV-1 bioactivity, especially genkwanine N (**69**, EC_50_: 0.17 nM and SI: 187,010) [8].

Based on the discovery of the antiretroviral activity of the dichloromethane extract of *D. gnidium* without displaying cytotoxicity, daphnetoxin (**56**), gnidicin (**16**), gniditrin (**20**), and excoecariatoxin (**58**) were determined to be the principles of anti-HIV bioactivity according to the HPLC-based profiling; meanwhile, a more detailed study showed that daphnetoxin (**56**) selectively interfered with the expression of two key cell-surface factors CXCR4 and CCR5 for HIV-1 entry [106].

It is worth noting that wikstroelide M (**77**) inhibited not only HIV-1 but HIV-2 strains in a concentration-dependent manner with high SI values and low cytotoxicity, and its mechanism might be related to the inhibition of HIV-1 reverse transcription and integrase nuclear translocation [71].

### 3.3. Anti-Inflammatory Activity

Genkwadaphnin (**15**), gniditrin (**20**), and yuanhuatine (**47**) showed significant inhibitory effect against LSP-induced nitric oxide (NO) production in RAW 264.7 macrophages with the IC_50_ values of 0.03–0.07 μM, especially 12-*O*-benzoylphorbol-13-nonanoate (**124**) and 12-*O*-benzoylphorbol-13-octanoate (**85**) with the IC_50_ values of 0.01 μM could be potential therapeutic agents for inflammation [20]. Daphwanin (**132**, IC_50_: 7.2 μM), a tigliane-type diterpenoid derived from *D. genkwa*, and orthobenzoate 2 (**64**, IC_50_: 5.4 μM) showed stronger inhibitory activity than aminoguanidine (IC_50_: 17.4 μM) on NO production in RAW 264.7 cells [67].

As for another inflammatory inhibitor genkwadaphnin (**15**), the mechanism includes the activation of PKD1/NF-κB signaling to induce CD44 expression in a time- and concentration-dependent manner and thus promoting the migration of K562 cells, resulting in an innate immune response [39]. Genkwadaphnin (**15**) was also observed to restore exhausted LCMV-specific CD4^+^ and CD8^+^ T cells by downregulating negative regulatory molecule Tim-3 [118].

It is reported that diterpenes showed anti-inflammatory efficacy sorted from highest to lowest as follows: prostratin Q (**128**), genkwadaphnin (**15**), isoyuanhuacine (**21**), yuanhuacine (**30**), yuanhuaoate C **(53**), yuanhuagine (**33**), isoyuanhuadine (**22**), and yuanhuadine (**31**) in LPS-induced RAW264.7 cells by downregulating the overexpression of IL-6, IL-1β, and TNF-α as well as decreasing NO production as the results of principal component analysis (PCA) and hierarchical cluster analysis (HCA) indicated, among these, prostratin Q (**128**) also showed bioactivity on VEGF, MMP-3, and ICAM, which has the potential to be developed as a novel drug for rheumatoid arthritis treatment [31].

### 3.4. Cholesterol-Lowering Activity

Gniditrin (**20**) and daphnetoxin (**56**) extracted from *D. giraldii* were found to present potent cholesterol-lowering activity in vitro at the EC_50_ levels of 0.59 and 4.3 μM, respectively, by up-regulating the low-density lipoprotein receptor (LDLR) level and consequently promoting LDLR expression [119]. Gniditrin (**20**) had a lower EC_50_ for activating LDLR-promoter than that of daphnetoxin (**56**), which might indicate that the acyl group at C-12 might improve the cholesterol-lowering bioactivity as well.

### 3.5. Neurotrophic Activity

Yuanhuacine (**30**) and genkwanine N (**69**) significantly enhanced the function of the orphan nuclear receptor Nurr1 at a concentration of 0.3 μM and inhibited LPS-induced neuroinflammation in vitro as well as improved behavioral deficits in a hydroxydopamine (6-OHDA) -induced rat model of Parkinson’s disease [17].

Dapholosericin A (**133**), a tigliane diterpene from the EtOAc extract of *D. holosericea*, was discovered to be a moderate acetylcholinesterase (AChE) inhibitor at a concentration of 100 μM, which suggested its potential usage in the mediation of Alzheimer’s disease [65].

Genkwalathins A (**134**) and B (**135**), two lathyrane-type diterpenes isolated from the chloroform extract from *D. genkwa*, were demonstrated to inhibit LPS-induced NO production in microglial BV-2 cells moderately (IC_50_: 43.08–46.77 μM) with nearly non-cytotoxicity, while yuanhuapine (**46**), yuanhuatine (**47**), genkwanine M (**68**), genkwanine N (**69**), yuanhuafine (**32**), and genkwadaphnin (**15**) showed much stronger anti-neuroinflammatory effect with the IC_50_ value below 0.44 μM, affecting cell viability slightly (17.5%–32.5% cell death at 10 μM) [66].

### 3.6. Antifertility Activity

The flowers of *D. genkwa* are also used as a traditional Chinese herbal remedy for abortion. Both yuanhuacine (**30**) and yuanhuadine (**31**) have already been used clinically as labor-induced drugs at the per capita dose of 70–80 and 60 μg, respectively [42]. Yuanhuatine (**47**) [56] and tanguticacine (**26**) [25] also showed antifertility activity in Rhesus monkeys at the dosage levels of 50 and 300 μg/monkey, respectively. Further experiments suggested that the hydroxyl group at C-15 and C-20 in diterpenoid orthoesters favored antifertility activity and esterification of the hydroxyl group at C-20 by long-chain fatty acid would decrease the toxicity of the diterpenoids [120], and the underlying mechanism was preliminarily established that these diterpenoids induced the release of endogenous prostaglandin by damaging decidual cells [13].

### 3.7. Skin Irritant, Nematicidal, and Piscicidal Activity

Many naturally occurring daphnane and tigliane diterpenoids, including huratoxin (**59**), mezerein (**24**), simplexin (**60**), and pimelea factor P_2_ (**99**), are highly skin irritant and toxic, thus considered as toxic principles in *Daphne* species [86,121]. The SAR involved is that a 12-acyloxy group favors irritancy [19].

Mezerein (**24**) was isolated from the seeds of *D. mezereum* and verified to be skin irritant just as daphnetoxin (**56**) [33]. Daphnegiraldifine (**55**), daphnetoxin (**56**), and 12-hydroxydaphnetoxin (**39**) obtained from *D. giraldii* were discovered to exert skin irritant activity [52]. Huratoxin (**59**), genkwadaphnin (**15**), prohuratoxin (**77**), and 1,2α-dihydrodaphnetoxin (**73**) were isolated from *D. feddei*, all of which have been determined to be skin irritant, and Huratoxin (**59**) exhibited the highest irritant activity among these four compounds [19].

It has been discovered that the benzene extract from the roots of *D. odora* was nematicidal to *Aphelenchoides besseyi*; then, further study showed that odoracin (**25**) had nematicidal activity towards *A. besseyi* was 100% (5 ppm) after 5 days [35]. Daphnetoxin (**56**) was reported to be a major poisonous component in *D. mezereum* [61]. Odoracin (**25**), along with odoratrin (**45**), gniditrin (**20**), 12-*O*-(E)-cinnamoyl-9,13,14-ortho-(2*E*,4*E*)-decadienylidyne-5β,12β-dihydroxyresiniferonol-6α,7α-oxide (**41**) and 12-*O*-(E)-cinnamoyl-9,13,14-ortho-(2*E*,4*E*,6*E*)-decatrienylidyne-5β,12β-dihydroxyresiniferonol-6α,7α-oxide (**40**), was found to have ornithine decarboxylase (ODC)-inducing activity in mouse skin, which correlated with a tumor-promoting effect; these compounds were simultaneously determined to be piscicidal [27]. 

## 4. Discussion

Back to 1970, the very first diterpenoid from *Daphne* species, daphnetoxin (**56**), was isolated as a toxic principle [61]. A total of 135 diterpenoids have been discovered from the genus *Daphne* after decades. These diterpenoids could be classified as daphnane-, tigliane-, and lathyrane-types according to the oxygen-containing functions and substitution pattern, and daphnane-type could be subdivided into 6-epoxy, genkwanine, 1-alkyldaphnane, and resiniferonoid. Within more and more novel diterpenoids isolated from *Daphne* genera, some of them could not fall into any classification. For example, genkwanine L (**95**) with a 3-ketone and 12-acetyl group does not completely satisfy the definition of genkwanines. As for compound **108**–**123**, one or more suitable classifications have not been established yet.

Although the genus of *Daphne* embraces more than ninety species distributed in Asia, Africa, and Europe [2], the origins of these diterpenoids are limited to fifteen *Daphne* species and diterpenoids have been reported to occur mainly in *D. genkwa*. For *D. mucronata*, only two isolated diterpenoids but one of those, gnidilatimonoein (**54**), showed potent anticancer activity [59,60,122,123], which suggests the potential in the diterpenoids from the genus *Daphne*. The distributions of every diterpenoid in different parts of plants are available in the hope that it would be a guide for isolation and identification of diterpenoids.

Furthermore, some *Daphne* species have been assayed and found to possess pharmacological activities which might be closely related to diterpenoids. The methanol extract of *D. malyana* showed significant antimicrobial potential [124] and the identification of the antibacterial or antifungal principles has not been reported yet. *D. linearifolia*, of which the stem bark was used to treat inflammation and rheumatism such as some *Daphne* species mentioned above, showed affinity towards a promising anticancer target Hsp90 [125]. The *D. cneorum* extract exhibits potent antimicrobial and antioxidant activities, which makes it a possible source of new agents [126]. For *D. alpina*, its antioxidant and antimicrobial bioactivities have been investigated [127,128] and gniditrin (**20**) has been isolated from this species [29]. These suggest a prospect of further phytochemistry and pharmacological study in *Daphne* species.

For the structure–activity relationship (SAR) summarized in this review, despite the fact that some of the SAR remains unclear and needs to be further investigated, it showed that the pharmacological effect, to a large extent, depends on the substituents at C-3, C-12, C-20, and C-1′ and the saturation of the ring A in these molecules. The SAR study is believed to provide very useful information for the optimization and synthesis of diterpenoids and lead to the development of novel agents; however, the SAR of diterpenoids has not been completely clarified.

## 5. Conclusions

In this review, a total of 135 natural diterpenoids in the past decades from the plants of the genus *Daphne* were covered. Many of the *Daphne* species are bioactive and thus have been used as traditional treatments for various diseases. Both the source species and parts from which diterpenoids were isolated have been provided as detailed reference information. The natural diterpenoids from *Daphne* species present interesting structures with complicated stereochemistry, which are closely related to their abundant bioactivities. The biological activities and the structure–activity relationship of certain classes of diterpenoids were reviewed in the hope of providing an easier way for researchers to understand the general situation of phytochemical and pharmacological properties in diterpenoids from the genus *Daphne*.

## Figures and Tables

**Table 1:** Structures and sources of 12-hydroxydaphnetoxins (**1**–**54**).

No.	Compound (Synonym)	Chemical Structure	Source Species (Part) ^1^
**1**	Acutilobin A	R_1_ = Ph, R_2_ = OCO(CH=CH)_2_CHOCH(CH_2_)_2_CH_3_, R_3_ = H	*D. acutiloba* (stems) [8]
**2**	Acutilobin B	R_1_ = Ph, R_2_ = OCO(CH=CH)_3_CH(OH)CH_2_CH_3_, R_3_ = H	*D. acutiloba* (stems) [8]
**3**	Acutilobin C	R_1_ = (CH$\overset{Z}{=}$CH) (CH=CH)_2_(CH_2_)_2_CH_3_, R_2_ = OCOCH=CHC_6_H_3_(3-OCH_3_) (4-OH), R_3_ = H	*D. acutiloba* (stems) [8]; *D. odora* (leaves and branches) [4]
**4**	Acutilobin D	R_1_ = (CH$\overset{Z}{=}$CH) (CH=CH) (CH_2_)_4_CH_3_, R_2_ = OCOCH=CHC_6_H_3_(3-OCH_3_) (4-OH), R_3_ = H	*D. acutiloba* (stems) [8]; *D. odora* (leaves and branches) [4]
**5**	Acutilobin E	R_1_ = Ph, R_2_ = OCOCH=CHC_6_H_3_(3-OCH_3_) (4-OH), R_3_ = H	*D. acutiloba* (stems) [8]
**6**	Altadaphnan C	R_1_ = Ph, R_2_ = OCO(CH$\overset{Z}{=}$CH) (CH=CH)_2_(CH_2_)_2_CH_3_, R_3_ = H	*D. altaica* (aerial parts) [9]
**7**	Daphgenkin F	R_1_ = Ph, R_2_ = OCO(CH_2_)_2_CH_3_, R_3_ = H	*D. genkwa* (buds) [10]
**8**	Daphgenkin G	R_1_ = Ph, R_2_ = OCOCH(CH_3_)_2_, R_3_ = H	*D. genkwa* (buds) [10]
**9**	Daphnegiraldicine	R_1_ = Ph, R_2_ = OCOCH=CH(CH_2_)_3_CH_3_, R_3_ = H	*D. giraldii* (stem bark) [11,12]
**10**	Daphnegiraldidine	R_1_ = Ph, R_2_ = OCO(CH_2_)_10_CH_3_, R_3_ = H	*D. giraldii* (stem bark) [12,13]
**11**	Daphneodorin D	R_1_ = (CH=CH)_2_(CH_2_)_4_CH_3_, R_2_ = OCOCH=CHC_6_H_4_(4-OH), R_3_ = H	*D. odora* (leaves and branches) [4]
**12**	Daphneodorin E	R_1_ = (CH=CH)_3_(CH_2_)_2_CH_3_, R_2_ = OCOCH=CHC_6_H_4_(4-OH), R_3_ = H	*D. odora* (leaves and branches) [4]
**13**	Genkwadane D	R_1_ = (CH=CH)_2_(CH_2_)_4_CH_3_, R_2_ = OCOCH(CH_3_)_2_, R_3_ = H	*D. genkwa* (buds) [10,14]
**14**	Genkwadaphin 20-palmitate	R_1_ = Ph, R_2_ = OBz, R_3_ = CO(CH_2_)_14_CH_3_	*D. oleoides* (stems) [15]
**15**	Genkwadaphnin (Daphne factor F_2_, 12-benzoyloxy daphnetoxin)	R_1_ = Ph, R_2_ = OBz, R_3_ = H	*D. genkwa* (buds [10], flos [16], roots and stems [17], roots [18]); *D. feddei* (roots) [19]; *D. oleoides* (stems) [15]; *D. aurantiaca* (stem bark) [20]; *D. altaica* (aerial parts) [9]
**16**	Gnidicin	R_1_ = Ph, R_2_ = OCOCH=CH–Ph, R_3_ = H	*D. papyracea* (NA ^2^) [13]; *D. giraldii* (stem bark) [21]; *D. tangutica* (root bark) [22]; *D. acutiloba* (stems) [8]; *D. gnidium* (aerial parts) [23]; *D. altaica* (aerial parts) [9]
**17**	Gnidicin 20-palmitate	R_1_ = Ph, R_2_ = OCOCH=CH–Ph, R_3_ = CO(CH_2_)_14_CH_3_	*D. oleoides* (stems) [15]
**18**	Gnididin	R_1_ = Ph, R_2_ = OCO(CH=CH)_2_(CH_2_)_4_CH_3_, R_3_ = H	*D. acutiloba* (stems) [8]
**19**	Gnidilatidin 20-palmitate	R_1_ = (CH=CH)_2_(CH_2_)_4_CH_3_, R_2_ = OBz, R_3_ = CO(CH_2_)_14_CH_3_	*D. oleoides* (stems) [15]; *D. genkwa* (buds) [24]
**20**	Gniditrin	R_1_ = Ph, R_2_ = CO(CH=CH)_3_(CH_2_)_2_CH_3_, R_3_ = H	*D. tangutica* (root bark [22,25], roots and stems [26]); *D. odora* (roots [27], leaves and branches [4]); *D. papyracea* (NA) [13]; *D. giraldii* (stem bark) [21]; *D. aurantiaca* (stems) [20]; *D. gnidium* (aerial parts) [23]; *D. acutiloba* (stems) [8]; *D. mezereum* (bark) [28,29]; *D. alpina* (bark) [29]
**21**	Isoyuanhuacine	R_1_ = (CH=CH) (CH$\overset{Z}{=}$CH) (CH_2_)_4_CH_3_, R_2_ = OBz, R_3_ = H	*D. genkwa* (buds) [30,31]
**22**	Isoyuanhuadine	R_1_ = (CH=CH) (CH$\overset{Z}{=}$CH) (CH_2_)_4_CH_3_, R_2_ = OAc, R_3_ = H	*D. genkwa* (buds) [10,14,30,31,32]
**23**	Kirkinine	R_1_ = (CH=CH)_3_(CH_2_)_2_CH_3_, R_2_ = OAc, R_3_ = H	*D. acutiloba* (stems) [8]
**24**	Mezerein	R_1_ = Ph, R_2_ = OCO(CH=CH)_2_–Ph, R_3_ = H	*D. mezereum* (seeds [33,34], barks [28])
**25**	Odoracin	R_1_ = (CH=CH) (CH$\overset{Z}{=}$CH) (CH_2_)_4_CH_3_, R_2_ = OBz, R_3_ = H	*D. odora* (roots) [27,35]
**26**	Tanguticacine	R_1_ = Ph, R_2_ = OCO(CH=CH)_3_(CH_2_)_2_CH_3_, R_3_ = CO(CH_2_)_14_CH_3_	*D. tangutica* (root bark) [12,25,36]
**27**	Tanguticadine	R_1_ = Ph, R_2_ = OCO(CH=CH)_3_(CH_2_)_2_CH_3_, R_3_ = 5,20-acetonide	*D. tangutica* (NA) [13,36]
**28**	Tanguticafine	R_1_ = Ph, R_2_ = OCOCH=CH–Ph, R_3_ = 5,20-acetonide	*D. tangutica* (NA) [13,36]
**29**	Tanguticagine	R_1_ = Ph, R_2_ = OCOCH=CH–Ph, R_3_ = CO(CH_2_)_14_CH_3_	*D. tangutica* (NA) [13,36]
**30**	Yuanhuacine (Gnidilatidin)	R_1_ = (CH=CH)_2_(CH_2_)_4_CH_3_, R_2_ = OBz, R_3_ = H	*D. genkwa* (roots [37], roots and stems [38], buds [31,39], flos [40]) *D. oleoides* (stems) [15]; *D. tangutica* (stems) [41]; *D. odora* (leaves and branches) [4]
**31**	Yuanhuadine	R_1_ = (CH=CH)_2_(CH_2_)_4_CH_3_, R_2_ = OAc, R_3_ = H	*D. genkwa* (roots and stems [38], roots [42], buds [30,31,43,44])
**32**	Yuanhuafine	R_1_ = Ph, R_2_ = OAc, R_3_ = H	*D. genkwa* (buds [14,44], flowers [45,46])
**33**	Yuanhuagine	R_1_ = (CH=CH)_3_(CH_2_)_2_CH_3_, R_2_ = OAc, R_3_ = H	*D. genkwa* (roots and flowers [47], buds [10,24,31,48], roots [48], flowers [49])
**34**	Yuanhuajine	R_1_ = (CH=CH)_3_(CH_2_)_2_CH_3_, R_2_ = OBz, R_3_ = H	*D. genkwa* (buds [10,48], roots and flowers [47]; roots [48]) *D. acutiloba* (stems) [8]; *D. tangutica* (stems) [41]; *D. odora* (leaves and branches) [4]
**35**	Yuanhuamine A	R_1_ = (CH$\overset{Z}{=}$CH)_2_(CH_2_)_4_CH_3_, R_2_ = OAc, R_3_ = H	*D. genkwa* (buds) [50]
**36**	Yuanhuamine B	R_1_ = (CH=CH)_2_(CH_2_)_4_CH_3_, R_2_ = OCO(CH_2_)_3_CH_3_, R_3_ = H	*D. genkwa* (buds) [50]
**37**	Yuanhuamine C	R_1_ = (CH=CH)_2_(CH_2_)_4_CH_3_, R_2_ = OCO(CH_2_)_4_CH_3_, R_3_ = H	*D. genkwa* (buds) [50]
**38**	Yuanhuaoate A	R_1_ = Ph, R_2_ = OCOCH_2_CH_3_, R_3_ = H	*D. genkwa* (buds) [51]
**39**	12-hydroxydaphnetoxin	R_1_ = Ph, R_2_ = OH, R_3_ = H	*D. giraldii* (roots [52], stem bark [11]); *D. genkwa* (buds) [10]
**40**	12-*O*-(E)-cinnamoyl-9,13,14-ortho-(2*E*,4*E*,6*E*)-decatrienylidyne-5β,12β-dihydroxyresiniferonol-6α,7α-oxide	R_1_ = (CH=CH)_3_(CH_2_)_2_CH_3_, R_2_ = OCOCH=CH–Ph, R_3_ = H	*D. odora* (roots [27], leaves and branches [4])
**41**	12-*O*-(E)-cinnamoyl-9,13,14-ortho-(2*E*,4*E*)-decadienylidyne-5β,12β-dihydroxyresiniferonol-6α,7α-oxide	R_1_ = (CH=CH)_2_(CH_2_)_4_CH_3_, R_2_ = OCOCH=CH–Ph, R_3_ = H	*D. odora* (roots [27], leaves and branches [4])
**42**	5β-hydroxyresiniferonol-6α,7α-epoxy-12β-acetoxy-9,13,14-ortho-2*E*-decenoate	R_1_ = (CH=CH) (CH_2_)_5_CH_3_, R_2_ = OAc, R_3_ = H	*D. genkwa* (flowers) [46]
**43**	Altadaphnan A	R = OCOCH=CH—Ph	*D. altaica* (aerial parts) [9]
**44**	Altadaphnan B	R = OCO(CH$\overset{Z}{=}$CH) (CH=CH)_2_(CH_2_)_2_CH_3_	*D. altaica* (aerial parts) [9]
**45**	Odoratrin	R = OCO(CH=CH)_3_(CH_2_)_2_CH_3_	*D. odora* (roots) [27]
**46**	Yuanhuapine	R = OAc	*D. genkwa* (buds [44,53,54], flos [46,55])
**47**	Yuanhuatine	R = OBz	*D. genkwa* (buds [14,56], flos [55], roots and stems [38]); *D. aurantiaca* (stem bark) [20]
**48**	Daphgenkin D	R_1_ = H, R_2_ = OAc	*D. genkwa* (buds) [10]
**49**	Daphgenkin E	R_1_ = CO(CH=CH)_3_(CH_2_)_2_CH_3_, R_2_ = OAc	*D. genkwa* (buds) [10]
**50**	Daphnane-type diterpene ester-7	R_1_ = CO(CH=CH)_2_(CH_2_)_4_CH_3_, R_2_ = OBz	*D. genkwa* (buds) [57,58]; *D. odora* (roots) [27]
**51**	Daphneodorin F	R_1_ = CO(CH=CH)_3_(CH_2_)_2_CH_3_, R_2_ = OCOCH=CHC_6_H_3_(3-OCH_3_)(4-OH)	*D. odora* (leaves and branches) [4]
**52**	Daphneodorin G	R_1_ = CO(CH=CH)_2_(CH_2_)_4_CH_3_, R_2_ = H, R_2_ = OCOCH=CH–Ph	*D. odora* (leaves and branches) [4]
**53**	Yuanhuaoate C	R_1_ = CO(CH=CH)_2_(CH_2_)_4_CH_3_, R_2_ = OAc	*D. genkwa* (buds) [10,31,51]
**54**	Gnidilatimonoein		*D. mucronata* (leaves) [59,60]

^1^ The fine lines in the table are used to separate the diterpenoids with slightly different skeletons (same in the tables below). ^2^ For the diterpenoids with multiple sources, the source species are listed in the chronological order in which the diterpenoids were isolated from them (same in the tables below).

**Table 2:** Structures and sources of daphnetoxins (**55**–**83**).

No.	Compound (Synonym)	Chemical Structure	Source Species (Part)
**55**	Daphnegiraldifine	R_1_ = Ph, R_2_ = H, R_3_ = CO(CH_2_)_14_CH_3_	*D. giraldii* (roots and stem bark) [52];
**56**	Daphnetoxin	R_1_ = Ph, R_2_ = H, R_3_ = H	*D. mezereum* (bark [28,61], seeds [33]); *D. papyracea* (NA) [13]; *D. giraldii* (roots and stem bark [52], roots and leaves [62], aerial parts [62], stems [21]); *D. tangutica* (root bark) [22,25]; *D. acutiloba* (stems) [8]; *D. gnidium* (stem bark [63], aerial parts [23]); *D. mucronata* (shoots) [64]; *D. altaica* (aerial parts) [9]
**57**	Excoecaria factor O_1_	R_1_ = (CH=CH)_3_(CH_2_)_2_CH_3_, R_2_ = H, R_3_ = H	*D. acutiloba* (stems) [8]; *D. tangutica* (stems) [41]
**58**	Excoecariatoxin	R_1_ = (CH=CH)_2_(CH_2_)_4_CH_3_, R_2_ = H, R_3_ = H	*D. tangutica* (root bark) [22,25]; *D. gnidium* (aerial parts) [23]; *D. altaica* (aerial parts) [9]
**59**	Huratoxin (Daphne factor F_1_)	R_1_ = (CH=CH)_2_(CH_2_)_8_CH_3_, R_2_ = H, R_3_ = H	*D. feddei* (roots) [19]
**60**	Simplexin	R_1_ = (CH_2_)_8_CH_3_, R_2_ = H, R_3_ = H	*D. genkwa* (buds) [14]; *D. holosericea* (stems) [65]
**61**	Tanguticaline	R_1_ = Ph, R_2_ = H, R_3_ = CO(CH_2_)_16_CH_3_	*D. tangutica* (NA) [13,36]
**62**	Tanguticamine	R_1_ = Ph, R_2_ = H, R_3_ = COCH=CH(CH_2_)_14_CH_3_	*D. tangutica* (NA) [13,36]
**63**	Yuanhuahine	R_1_ = (CH=CH)_2_(CH_2_)_4_CH_3_, R_2_ = CH_2_CH_3_, R_3_ = H	*D. genkwa* (flowers [49], buds [10,14,43])
**64**	Yuanhualine	R_1_ = (CH=CH)_2_(CH_2_)_4_CH_3_, R_2_ = CH_2_CH_2_CH_3_, R_3_ = H	*D. genkwa* (flowers [49], buds [10,44])
**65**	14′-ethyltetrahydrohuratoxin	R_1_ = (CH_2_)_14_CH_3_, R_2_ = H, R_3_ = H	*D. acutiloba* (stems) [8]
**66**	Acutilobin F	R_1_ = OCO(CH=CH)_3_(CH_2_)_2_CH_3_, R_2_ = H	*D. acutiloba* (stems) [8]; *D. genkwa* (roots and stems) [38]
**67**	Acutilobin G	R_1_ = OCOCH=CH–Ph, R_2_ = H	*D. acutiloba* (stems) [8]
**68**	Genkwanine M	R_1_ = OH, R_2_ = Bz	*D. genkwa* (buds [14,54,57], flowers [66])
**69**	Genkwanine N	R_1_ = OBz, R_2_ = H	*D. genkwa* (buds [54], flowers [55,66], roots and stems [17])
**70**	Genkwanine N 20-palmitate	R_1_ = OBz, R_2_ = CO(CH_2_)_14_CH_3_	*D. genkwa* (buds) [24]
**71**	Orthobenzoate 2	R_1_ = OH, R_2_ = H	*D. genkwa* (buds [14,54,67], flowers [66])
**72**	Wikstroemia factor M_1_	R_1_ = OCO(CH=CH)_2_(CH_2_)_4_CH_3_, R_2_ = H	*D. acutiloba* (stems) [8]; *D. genkwa* (buds [68], roots and stems [38], flowers [55])
**73**	1,2α-dihydrodaphnetoxin (Tanguticakine, Daphne factor F_4_)	R_1_ = O, R_2_ = H	*D. feddei* (stem bark) [19]; *D. oleoides* (stems) [15]; *D. giraldii* (stems) [62]; *D. genkwa* (flowers) [66]
**74**	1,2α-dihydro-20-palimoyldaphnetoxin (1,2-dihydrodaphnegiraldifine)	R_1_ =O, R_2_ = CO(CH_2_)_14_CH_3_	*D. tangutica* (root bark) [13,69]
**75**	Daphnediraldigin	R_1_ = Bz, R_2_ = H	*D. giraldii* (stem bark) [70]
**76**	Isovesiculosin	R_1_ = H, R_2_ = CO(CH=CH)_2_(CH_2_)_4_CH_3_	*D. tangutica* (root bark) [22]
**77**	Prohuratoxin (Wikstroelide M, Daphne factor F_3_)	R_1_ = CO(CH=CH)_2_(CH_2_)_8_CH_3_, R_2_ = H	*D. feddei* (roots) [19]; *D. acutiloba* (stems) [71]; *D. altaica* (aerial parts) [9]
**78**	Vesiculosin	R_1_ = CO(CH=CH)_2_(CH_2_)_4_CH_3_, R_2_ = H	*D. tangutica* (root bark) [22]; *D. altaica* (aerial parts) [9]
**79**	15,16-dihydrodaphnetoxin (Tanguticahine)		*D. tangutica* (NA) [12,13,36]
**80**	1,2α-dihydro-5β-hydroxy-6α,7α-epoxy-resiniferonol-14-benzonate	R = β-CH_3_	*D. tangutica* (root bark) [22]
**81**	1,2β-dihydro-5β-hydroxy-6α,7α-epoxy-resiniferonol-14-benzoate	R = α-CH_3_	*D. tangutica* (root bark) [22]
**82**	Genkwanin I		*D. genkwa* (buds [72], flos [66])
**83**	Genkwanine O		*D. genkwa* (buds [54], flos [66])

**Table 3:** Structures and sources of genkwanines (**84**–**95**).

No.	Compound (Synonym)	Chemical Structure	Source Species (Part)
**84**	Genkwanine A	R_1_ = H, R_2_ = H	*D. genkwa* (buds [43,57,73], flos [66])
**85**	Genkwanine B	R_1_ = CO(CH=CH)_2_(CH_2_)_4_CH_3_, R_2_ = H	*D. genkwa* (buds) [73]
**86**	Genkwanine C	R_1_ = CO(CH=CH)_3_(CH_2_)_2_CH_3_, R_2_ = H	*D. genkwa* (buds) [73]
**87**	Genkwanine D	R_1_ = Bz, R_2_ = H	*D. genkwa* (buds) [54,57,73]
**88**	Genkwanine E	R_1_ = H, R_2_ = CO(CH=CH)_3_(CH_2_)_2_CH_3_	*D. genkwa* (buds) [73]
**89**	Genkwanine F	R_1_ = H, R_2_ = CO(CH=CH)_2_(CH_2_)_4_CH_3_	*D. genkwa* (buds [14,43,54,73], flowers [55])
**90**	Genkwanine G	R_1_ = H, R_2_ = CO(CH=CH) (CH_2_)_6_CH_3_	*D. genkwa* (buds) [73]
**91**	Genkwanine H	R_1_ = H, R_2_ = Bz	*D. genkwa* (buds [43,54,57,73], flowers [67])
**92**	Genkwanine I	R = H	*D. genkwa* (buds [73,74], flowers [67])
**93**	Genkwanine J	R = CO(CH=CH)_2_(CH_2_)_4_CH_3_	*D. genkwa* (buds) [14,57,73,74]
**94**	Genkwanine K	R = Bz	*D. genkwa* (buds [73], flowers [66])
**95**	Genkwanine L		*D. genkwa* (buds [73], flowers [46])

**Table 4:** Structures and sources of resiniferonoids (**96**–**97**).

No.	Compound (Synonym)	Chemical Structure	Source Species (Part)
**96**	Daphneresiniferin A	R = OAc	*D. genkwa* (buds) [43]
**97**	Daphneresiniferin B	R = OBz	*D. genkwa* (buds) [43]

**Table 5:** Structures and sources of 1-alkyldaphnanes (**98**–**107**).

No.	Compound (Synonym)	Source Species (Part)
**98**	Wikstroelide E	*D. genkwa* (buds) [14]
**99**	Pimelea factor P_2_	*D. genkwa* (buds) [14]
**100**	Genkwadane B	*D. genkwa* (buds) [14]
**101**	Gnidimacrin	*D. odora* (branches) [76]
**102**	Daphneodorin A	*D. odora* (branches) [76]
**103**	Daphneodorin B	*D. odora* (branches) [76]
**104**	Daphneodorin C	*D. odora* (branches) [76]
**105**	Pimelotide A	*D. genkwa* (buds) [14,44]
**106**	Pimelotide C	*D. genkwa* (buds) [14]
**107**	Genkwadane C	*D. genkwa* (buds) [14]

**Table 6:** Structures and sources of other daphnane diterpenoids from the genus *Daphne* (**108**–**123**).

No.	Compound (Synonym)	Chemical Structure	Source Species (Part)
**108**	Daphgenkin A		*D. genkwa* (buds) [10]
**109**	Daphgenkin B	R = OBz	*D. genkwa* (buds) [10]
**110**	Daphgenkin C	R = OAc	*D. genkwa* (buds) [10]
**111**	Genkwadane A		*D. genkwa* (buds) [14]
**112**	Yuanhuaoate B		*D. genkwa* (buds) [51]
**113**	Daphneodorin H	R_1_ = H, R_2_ = CO(CH=CH)_3_(CH_2_)_2_CH_3_, R_3_ = H, R_4_ = Bz	*D. odora* (leaves and branches) [4]
**114**	Genkwanine VIII	R_1_ = Bz, R_2_ = H, R_3_ = H, R_4_ = Bz	*D. genkwa* (flowers) [44,84]
**115**	Neogenkwanine A	R_1_ = Bz, R_2_ = H, R_3_ = H, R_4_ = H	*D. genkwa* (buds) [57,85]
**116**	Neogenkwanine B	R_1_ = H, R_2_ = H, R_3_ = H, R_4_ = Bz	*D. genkwa* (buds) [57,85]
**117**	Neogenkwanine C	R_1_ = Bz, R_2_ = CO(CH=CH) (CH$\overset{Z}{=}$CH) (CH_2_)_4_CH_3_, R_3_ = H, R_4_ = H	*D. genkwa* (buds) [57,85]
**118**	Neogenkwanine D	R_1_ = Bz, R_2_ = CO(CH=CH)_2_(CH_2_)_4_CH_3_, R_3_ = H, R_4_ = H	*D. genkwa* (buds) [57,85]
**119**	Neogenkwanine E	R_1_ = H, R_2_ = CO(CH=CH)_2_(CH_2_)_4_CH_3_, R_3_ = H, R_4_ = Bz	*D. genkwa* (buds) [57,74,85]
**120**	Neogenkwanine F	R_1_ = H, R_2_ = CO(CH=CH) (CH$\overset{Z}{=}$CH) (CH_2_)_4_CH_3_, R_3_ = H, R_4_ = Bz	*D. genkwa* (buds) [57,74,85]
**121**	Neogenkwanine G	R_1_ = Bz, R_2_ = CO(CH=CH) (CH$\overset{Z}{=}$CH) (CH_2_)_4_CH_3_, R_3_ = Bz, R_4_ = Bz	*D. genkwa* (buds) [57]
**122**	Neogenkwanine I	R_1_ = H, R_2_ = H, R_3_ = Bz, R_4_ = H	*D. genkwa* (buds) [74,85]
**123**	Neogenkwanine H		*D. genkwa* (buds) [57]

**Table 7:** Structures and sources of tigliane-type diterpenoids (**124**–**133**).

No.	Compound (Synonym)	Chemical Structure	Source Species (Part)
**124**	12-*O*-benzoylphorbol-13-nonanoate	R_1_ = (CH_2_)_7_CH_3_, R_2_ = OBz	*D. aurantiaca* (stem bark) [20]
**125**	12-*O*-benzoylphorbol-13-octanoate	R_1_ = (CH_2_)_6_CH_3_, R_2_ = OBz	*D. aurantiaca* (stem bark [20], stems [87])
**126**	12-*O*-decanoylphorbol-13-acetate	R_1_ = CH_3_, R_2_ = OCO(CH_2_)_8_CH_3_	*D. genkwa* (buds) [85,92]
**127**	12-*O*-n-deca-2,4,6-trienoyl-phorbol-(13)-acetate	R_1_ = CH_3_, R_2_ = OCO(CH=CH)_3_(CH_2_)_2_CH_3_	*D. genkwa* (roots and stems) [38]
**128**	Prostratin Q (12-*O*-(2′*E*,4′*E*-decadienoyl)-4-hydroxyphorbol-13-acetyl)	R_1_ = CH_3_, R_2_ = OCO(CH=CH)_2_(CH_2_)_4_CH_3_	*D. genkwa* (buds [31,32,85], roots and stems [38,92])
**129**	Prostratin	R_1_ = CH_3_, R_2_ = H	*D. acutiloba* (stems) [8]
**130**	Phorbol 13-monoacetate	R_1_ = CH_3_, R_2_ = OH	*D. aurantiaca* (stems) [87]
**131**	12-*O*-(2′*E*,4′*E*-decadienoyl)-7-oxo-5-ene-phorbol-13-acetate	R_1_ = CH_3_, R_2_ = OCO(CH=CH)_2_(CH_2_)_4_CH_3_	*D. genkwa* (buds) [85,92]
**132**	Daphwanin (12-*O*-decanoyl-7-oxo-5-ene-phorbol-13-acetate)	R_1_ = CH_3_, R_2_ = OBz	*D. genkwa* (buds) [66,92]
**133**	Dapholosericin A	R_1_ = (CH_2_)_6_CH_3_, R_2_ = OBz	*D. holosericea* (stems) [65]

**Table 8:** Structures and sources of lathyrane-type diterpenoids (**134**–**135**).

No.	Compound (Synonym)	Source Species (Part)
**134**	Genkwalathin A	*D. genkwa* (flowers) [66]
**135**	Genkwalathin B	*D. genkwa* (flowers) [66]

## Data Availability

The data presented in this study are available in the Supplementary Materials.

## Supplementary Materials

The following are available online, Table S1: The IC_50_ values (μM) of cytotoxicity of some diterpenoids in various carcinoma cell lines in vitro.

## Abbreviations

*A. besseyi*	*Aphelenchoides besseyi* Chrisite
Ac	acetyl
AChE	acetylcholinesterase
AZT	3′-azido-3′-deoxythymidine
Bz	benzoyl
*D. acutiloba*	*Daphne acutiloba* Rehd.
*D. altaica*	*Daphne altaica* Pall.
*D. cneorum*	*Daphne cneorum* L.
*D. alpina*	*Daphne alpina* L.
*D. aurantiaca*	*Daphne aurantiaca* Diels.
*D. feddei*	*Daphne feddei* Lévl.
*D. genkwa*	*Daphne genkwa* Sieb. et Zucc.
*D. giraldii*	*Daphne giraldii* Nitsche
*D. gnidium*	*Daphne gnidium* L.
*D. holosericea*	*Daphne holosericea* (Diels) Hamaya
*D. linearifolia*	*Daphne linearifolia* Hart
*D. malyana*	*Daphne Malyana* Blečić
*D. mezereum*	*Daphne mezereum* L.
*D. mucronata*	*Daphne mucronata* Royle
*D. odora*	*Daphne odora* Thunb.
*D. oleoides*	*Daphne oleoides* Schreb.
*D. papyracea*	*Daphne papyracea* Wall. ex Steud.
*D. tangutica*	*Daphne tangutica* Maxim.
*D. laureola*	*Daphne laureola* L.
*E. resinifera*	*Euphorbia resinifera* O. Berg
*E. lathyris*	*Euphorbia lathyris* L.
EtOAc	ethyl acetate
LDLR	low-density lipoprotein receptor
NO	nitric oxide
ODC	ornithine decarboxylase
Ph	phenyl
PKC	protein kinase C
SAR	structure–activity relationship
Top I	DNA topoisomerase I
