# Supplementary material for: Phytochemistry and Pharmacological Activities of the Diterpenoids from the Genus Daphne
Source: Molecules. 2021 Oct 31;26(21):6598. doi: 10.3390/molecules26216598 (PMC8588408; doi:10.3390/molecules26216598)
Supplement: Supplementary file 1 [file molecules-26-06598-s001.zip › molecules-1420741-supplementary.pdf]

Table S1. The IC_{50} values (μM) of cytotoxicity of some diterpenoids in various carcinoma cell lines *in vitro*.

Compound	Leukemia		Hepatoma				Lung carcinoma					Breast carcinoma	
	HL−60	K562	SMMC−7721	HepG2	Hep3B	PLC/RPF/5	A549	H358	H1299	H1993	SK−MES−1	MCF−7	SK−BR−3
**1**	15.48 [8]	—	20.02 [8]	—	—	—	11.96 [8]	—	—	—	—	16.57 [8]	—
**2**	14.51 [8]	—	11.77 [8]	—	—	—	9.70 [8]	—	—	—	—	16.16 [8]	—
**3**	9.70 [8]	—	12.35 [8]	—	—	—	4.14 [8]	—	—	—	—	9.94 [8]	—
**4**	10.86 [8]	—	12.76 [8]	—	—	—	7.75 [8]	—	—	—	—	14.01 [8]	—
**5**	12.16 [8]	—	14.75 [8]	—	—	—	10.52 [8]	—	—	—	—	16.77 [8]	—
**6**	—	—	—	—	—	—	0.82	—	—	—	—	—	—
**13**	29.99 [14]	5.11 [14]	—	—	—	—	23.05 [14]	—	—	—	—	20.80 [14]	—
**15**	15.73 [8]	22.53 [14]	15.98 [8]	19.09 [14]	31.24 [44]	37.81 [44]	10.35 [8]	—	—	—	—	16.54 [8]	—
**18**	14.51 [8]	—	15.48 [8]	—	—	—	11.04 [8]	—	—	—	—	16.79 [8]	—
**21**	—	—	—	—	—	—	—	—	—	—	—	—	0.217 [21]
**22**	17.72 [14]	17.54 [14]	—	0.64 [14]	—	—	24.12 [14]	—	—	—	—	23.45 [14]	0.084 [21]
**30**	10.80 [39]	16.08 [14]	—	5.56 [14]	—	—	13.11 [14]	16.50 [105]	4.00 [105]	0.009 [105]	—	2.39 [14]	0.172 [21]
**31**	30.05 [14]	22.16 [14]	—	17.06 [14]	21.00 [44]	70.91 [44]	31.49 [44]	9.10 [128]	5.60 [128]	0.0047 [128]	0.000001 [128]	4.11 [14]	0.062 [21]
**32**	—	—	—	42.37 [14]	69.26 [44]	—	67.83 [44]	—	—	—	—	—	—
**33**	—	—	—	—	—	—	0.024 [106]	9.00 [106]	0.0051 [106]	0.074 [106]	0.022 [106]	—	0.809 [21]
**34**	14.05 [8]	—	38.11 [8]	—	—	—	21.35 [8]	—	—	—	—	16.64 [8]	—
**43**	—	—	—	—	—	—	0.24 [9]	—	—	—	—	—	—
**44**	—	—	—	—	—	—	0.28 [9]	—	—	—	—	—	—
**46**		—	—	50.00 [14]	—	56.34 [44]	65.72 [44]	—	—	—	—	23.62 [14]	—
**47**	26.02 [14]	24.64 [14]	—	20.31 [14]	—	—	12.46 [14]	—	—	—	—	0.37 [14]	0.17 [21]
**50**	18.51 [14]	29.03 [14]	—	16.67 [14]	—	—	13.34 [14]	—	—	—	—	—	—
**54**	0.0013 [59]	0.00094 [59]	—	—	—	—	—	—	—	—	—	—	—
**56**	15.73 [8]	—	38.73 [8]	—	—	—	18.47 [8]	—	—	—	—	—	—
**57**	17.60 [8]	—	26.75 [8]	—	—	—	12.55 [8]	—	—	—	—	19.42 [8]	—
**58**	12.76 [8]	—	13.83 [8]	—	—	—	10.35 [8]	—	—	—	—	16.10 [8]	—
**60**	—	—	—	21.72 [14]	—	—	26.96 [14]	—	—	—	—	25.41 [14]	—
**63**	22.70 [14]	29.35 [14]	—	25.52 [14]	—	—	18.78 [14]	0.50 [106]	0.0062 [106]	0.035 [106]	0.017 [106]	—	—

Table S1. *Continued*

Compound	Leukemia		Hepatoma				Lung carcinoma					Breast carcinoma	
	HL−60	K562	SMMC−7721	HepG2	Hep3B	PLC/RPF/5	A549	H358	H1299	H1993	SK−MES−1	MCF−7	SK−BR−3
**64**	—	—	—	—	69.40 [44]	56.88 [44]	98.46 [44]	4.70 [106]	0.0037 [106]	0.065 [106]	0.013 [106]	34.57 [44]	—
**65**	13.18 [8]	—	17.89 [8]	—	—	—	9.10 [8]	—	—	—	—	24.99 [8]	—
**66**	14.99 [8]	—	14.51 [8]	—	—	—	11.40 [8]	—	—	—	—	17.95 [8]	—
**67**	16.24 [8]	—	15.73 [8]	—	—	—	11.04 [8]	—	—	—	—	16.78 [8]	—
**68**	30.85 [14]	20.16 [14]	—	—	—	—	23.38 [14]	—	—	—	—	25.28 [14]	—
**69**	—	—	—	20.17 [14]	—	—	—	—	—	—	—	—	—
**71**	—	42.31 [14]	—	31.71 [14]	—	—	—	—	—	—	—	12.36 [14]	—
**72**	13.83 [8]	—	14.28 [8]	—	—	—	12.35 [8]	—	—	—	—	18.10 [8]	—
**89**	20.50 [14]	24.19 [14]	—	26.17 [14]	—	—	22.29 [14]	—	—	—	—	—	—
**93**	10.06 [14]	24.54 [14]	—	21.05 [14]	—	—	20.91 [14]	—	—	—	—	—	—
**98**	22.11 [14]	24.04 [14]	—	17.06 [14]	—	—	19.26 [14]	—	—	—	—	—	—
**99**	17.86 [14]	25.18 [14]	—	18.30 [14]	—	—	17.03 [14]	—	—	—	—	—	—
**100**	18.08 [14]	18.69 [14]	—	28.14 [14]	—	—	18.78 [14]	—	—	—	—	19.91 [14]	—
**101**	0.00028 [78]	0.00025 [78]	—	—	—	—	—	—	—	—	—	—	—
**105**	—	—	—	—	43.83 [44]	78.04 [44]	35.66 [14]	—	—	—	—	57.28 [44]	—
**106**	34.08 [14]	26.28 [14]	—	23.25 [14]	—	—	—	—	—	—	—	7.36 [14]	—
**107**	—	—	—	25.15 [14]	—	—	44.99 [14]	—	—	—	—	—	—
**111**	27.70 [14]	46.88 [14]	—	24.76 [14]	—	—	26.95 [14]	—	—	—	—	33.70 [14]	—
**112**	27.03 [14]	21.31 [14]	—	20.97 [14]	—	—	15.91 [14]	—	—	—	—	0.37 [14]	—
**114**	—	—	—	—	—	67.33 [44]	45.56 [44]	—	—	—	—	58.95 [44]	—
**117**	10.59 [57]	—	—	—	8.35 [57]	—	—	—	—	—	—	—	—
**118**	10.07 [57]	—	—	—	8.16 [57]	—	—	—	—	—	—	—	—
**119**	11.17 [57]	—	—	—	7.61 [57]	—	—	—	—	—	—	—	—
**120**	12.26 [57]	—	—	—	17.61 [57]	—	—	—	—	—	—	—	—

Table S1. *Continued*

Compound	Melanoma			Cervical carcinoma	Fibrosarcoma	Colon carcinoma						Glioma		Esophageal carcinoma
	A375—S2	B16	A2058	HeLa	HT1080	SW480	HCT116	COLO 205	HT—29	SW620	RKO	U251	U87	TE—1
**1**	—	—	—	—	—	13.34 [8]	—	—	—	—	—	—	—	—
**2**	—	—	—	—	—	15.30 [8]	—	—	—	—	—	—	—	—
**3**	—	—	—	—	—	12.93 [8]	—	—	—	—	—	—	—	—
**4**	—	—	—	—	—	15.07 [8]	—	—	—	—	—	—	—	—
**5**	—	—	—	—	—	14.68 [8]	—	—	—	—	—	—	—	—
**6**	—	—	—	—	—	—	—	—	—	—	—	—	—	—
**13**	24.87 [14]	—	—	—	4.55 [14]	—	25.15 [14]	—	—	—	—	—	—	—
**15**	16.88 [14]	—	—	19.09 [14]	0.10 [14]	16.03 [8]	29.28 [14]	—	—	—	—	54.37 [44]	—	27.05 [44]
**18**	—	—	—	—	—	15.61 [8]	—	—	—	—	—	—	—	—
**21**	—	—	—	—	—	—	—	—	—	—	—	—	—	—
**22**	21.52 [14]	—	—	0.64 [14]	14.89 [14]	—	32.31 [14]	—	—	—	—	—	—	—
**30**	8.72 [14]	18.00 [84]	15.00 [84]	5.56 [14]	2.02 [14]	—	22.96 [14]	3.00 [84]	23.00 [84]	4.30 [10]	6.50 [10]	—	—	—
**31**	15.75 [14]	11.00 [84]	13.00 [84]	17.06 [14]	0.10 [14]	—	32.65 [14]	2.00 [84]	13.00 [84]	—	—	62.14 [44]	—	49.82 [44]
**32**	—	—	—	42.37 [14]	0.10 [14]	—	37.53 [14]	—	—	—	—	—	—	77.76 [44]
**33**	—	—	—	—	—	—	—	—	—	—	—	—	—	—
**34**	—	—	—	—	—	15.07 [8]	—	—	—	—	—	—	—	—
**43**	—	—	—	—	—	—	—	—	—	—	—	—	—	—
**44**	—	—	—	—	—	—	—	—	—	—	—	—	—	—
**46**	14.24 [14]	—	—	50.00 [14]	0.10 [14]	—	—	—	—	—	—	58.58 [44]	—	69.67 [44]
**47**	9.31 [14]	—	—	20.31 [14]	14.35 [14]	—	24.87 [14]	—	—	—	—	—	—	—
**50**	26.51 [14]	—	—	16.67 [14]	18.40 [14]	—	16.44 [14]	—	—	—	—	—	—	—
**54**	—	—	—	—	—	—	—	—	—	—	—	—	—	—
**56**	—	—	—	—	—	—	—	—	—	—	—	—	—	—
**57**	—	—	—	—	—	17.70 [8]	—	—	—	—	—	—	—	—
**58**	—	—	—	—	—	14.67 [8]	—	—	—	—	—	—	—	—
**60**	10.65 [14]	—	—	21.72 [14]	0.10 [14]	—	—	—	—	—	—	—	—	—
**63**	—	28.00 [43]	—	25.52 [14]	28.80 [14]	—	46.79 [14]	—	—	—	—	—	—	—
**64**	—	—	—	—	—	—	—	—	—	5.20 [10]	9.70 [10]	56.23 [44]	—	56.89 [44]

Table S1. *Continued*

Compound	Melanoma			Cervical carcinoma	Fibrosarcoma	Colon carcinoma						Glioma		Esophageal carcinoma
	A375—S2	B16	A2058	HeLa	HT1080	SW480	HCT116	COLO 205	HT—29	SW620	RKO	U251	U87	TE—1
**65**	—	—	—	—	—	19.12 [8]	—	—	—	—	—	—	—	—
**66**	—	—	—	—	—	16.73 [8]	—	—	—	—	—	—	—	—
**67**	—	—	—	—	—	14.21 [8]	—	—	—	—	—	—	—	—
**68**	3.62 [14]	—	—	20.17 [14]	0.10 [14]	—	28.14 [14]	—	—	—	—	—	—	—
**69**	47.23 [14]	—	—	—	—	—	—	—	—	—	—	—	—	—
**71**	—	—	—	31.71 [14]	24.49 [14]	—	49.86 [14]	—	—	—	—	—	—	—
**72**	—	—	—	—	—	15.70 [8]	—	—	—	—	—	—	—	—
**89**	25.41 [14]	—	—	26.17 [14]	29.94 [14]	—	27.43 [14]	—	—	—	—	—	—	—
**93**	23.69 [14]	—	—	21.05 [14]	22.91 [14]	—	35.77 [14]	—	—	—	—	—	—	—
**98**	29.03 [14]	—	—	17.06 [14]	15.14 [14]	—	29.93 [14]	—	—	—	—	—	—	—
**99**	25.67 [14]	—	—	18.30 [14]	13.22 [14]	—	19.59 [14]	—	—	—	—	—	—	—
**100**	29.36 [14]	—	—	—	9.56 [14]	—	28.92 [14]	—	—	—	—	—	—	—
**101**	—	—	—	—	—	—	—	—	—	—	—	—	—	—
**105**	—	—	—	28.14 [14]	0.83 [14]	—	35.98 [14]	—	—	—	—	61.09 [44]	—	64.69 [44]
**106**	22.98 [14]	—	—	23.25 [14]	20.79 [14]	—	26.49 [14]	—	—	—	—	—	—	—
**107**	28.64 [14]	—	—	25.15 [14]	7.71 [14]	—	—	—	—	—	—	—	—	—
**111**	14.29 [14]	—	—	24.76 [14]	16.10 [14]	—	—	—	—	—	—	—	—	—
**112**	23.34 [14]	—	—	20.97 [14]	0.59 [14]	—	26.47 [14]	—	—	8.50 [10]	—	—	—	—
**114**	—	—	58.00 [84]	—	—	—	—	34.00 [84]	29.00 [84]	—	—	56.99 [44]	—	65.79 [44]
**117**	—	—	—	—	—	—	—	—	—	—	—	—	27.11 [57]	—
**118**	—	—	—	—	—	—	—	—	—	—	—	—	30.80 [57]	—
**119**	—	—	—	—	—	—	—	—	—	—	—	—	47.60 [57]	—
**120**	—	—	—	—	—	—	—	—	—	—	—	—	58.60 [57]	—
